# Three-way contact analysis characterizes the higher order organization of the *Tcra* locus

**DOI:** 10.1093/nar/gkad641

**Published:** 2023-08-03

**Authors:** Ranran Dai, Yongchang Zhu, Zhaoqiang Li, Litao Qin, Nan Liu, Shixiu Liao, Bingtao Hao

**Affiliations:** Cancer Research Institute, School of Basic Medical Sciences, Southern Medical University, Guangzhou, Guangdong Province 510515, China; Department of Immunology, School of Basic Medical, Zhengzhou University, Zhengzhou 450001, China; Medical Genetic Institute of Henan Province, Henan Key Laboratory of Genetic Diseases and Functional Genomics, National Health Commission Key Laboratory of Birth Defects Prevention, Henan Provincial People’s Hospital, People’s Hospital of Zhengzhou University, Zhengzhou University, Zhengzhou, Henan Province 450003, China; Cancer Research Institute, School of Basic Medical Sciences, Southern Medical University, Guangzhou, Guangdong Province 510515, China; Medical Genetic Institute of Henan Province, Henan Key Laboratory of Genetic Diseases and Functional Genomics, National Health Commission Key Laboratory of Birth Defects Prevention, Henan Provincial People’s Hospital, People’s Hospital of Zhengzhou University, Zhengzhou University, Zhengzhou, Henan Province 450003, China; Division of Obstetrics and Gynecology, Nanfang Hospital, Southern Medical University, Guangzhou, Guangdong Province 510515, China; Medical Genetic Institute of Henan Province, Henan Key Laboratory of Genetic Diseases and Functional Genomics, National Health Commission Key Laboratory of Birth Defects Prevention, Henan Provincial People’s Hospital, People’s Hospital of Zhengzhou University, Zhengzhou University, Zhengzhou, Henan Province 450003, China; Cancer Research Institute, School of Basic Medical Sciences, Southern Medical University, Guangzhou, Guangdong Province 510515, China; Department of Immunology, School of Basic Medical, Zhengzhou University, Zhengzhou 450001, China

## Abstract

The generation of highly diverse antigen receptors in T and B lymphocytes relies on V(D)J recombination. The enhancer E_α_ has been implicated in regulating the accessibility of V_α_ and J_α_ genes through long-range interactions during rearrangements of the T-cell antigen receptor gene *Tcra*. However, direct evidence for E_α_ physically mediating the interaction of V_α_ and J_α_ genes is still lacking. In this study, we utilized the 3C-HTGTS assay, a chromatin interaction technique based on 3C, to analyze the higher order chromatin structure of the *Tcra* locus. Our analysis revealed the presence of sufficient information in the 3C-HTGTS data to detect multiway contacts. Three-way contact analysis of the *Tcra* locus demonstrated the co-occurrence of the proximal J_α_ genes, V_α_ genes and E_α_ in CD4^+^CD8^+^ double-positive thymocytes. Notably, the INT2–TEAp loop emerged as a prominent structure likely to be responsible for bringing the proximal J_α_ genes and the V_α_ genes into proximity. Moreover, the enhancer E_α_ utilizes this loop to establish physical proximity with the proximal V_α_ gene region. This study provides insights into the higher order chromatin structure of the *Tcra* locus, shedding light on the spatial organization of chromatin and its impact on V(D)J recombination.

## Graphical Abstract

**Graphical Abstract:**
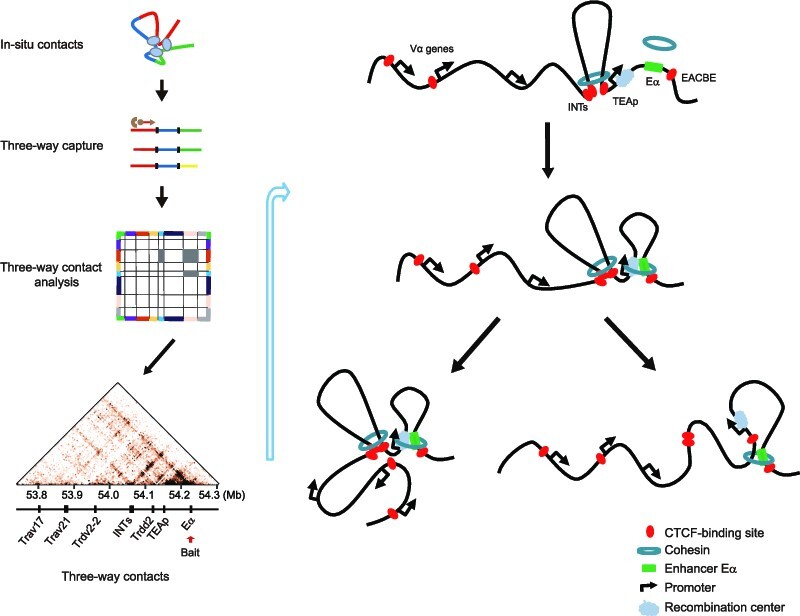

## INTRODUCTION

During the development of T and B lymphocytes, a high diversity of antigen receptors is generated through V(D)J recombination, which rearranges variable (V), joining (J) and, in some cases, diversity (D) gene segments in a nearly random fashion (1). Usually, V segments of antigen receptor genes are located far away from D and J segments, necessitating spatial proximity for rearrangement (1). Numerous studies have demonstrated the involvement of chromatin organizers (such as CTCF and cohesin) and *cis*-regulatory elements in antigen receptor loci in regulating the repertoire of immunoglobulins and T-cell receptors (TCRs) (2–11). Recently, it has been proposed that the recombinase RAG scans upstream chromatin, facilitated by cohesin-mediated loop extrusion, which plays a vital role in *Igh* rearrangement (6,10,12). This highlights the crucial role of chromatin organization in the rearrangement of antigen receptor genes.

The recombination of the T-cell receptor α gene (*Tcra*) is a crucial event during T-cell development in the thymus (13). *Tcra* recombination begins with rearrangement of the proximal V_α_ and the proximal J_α_ gene segments, referred to as primary rearrangement. The presence of the *Tcrd* gene and insulators creates a barrier for achieving spatial proximity between the V_α_ and J_α_ gene segments (2,13,14). Chen *et al.* reported that two CTCF-binding sequences called INT1 and INT2 (INTs), located between the V and DJ_δ_ segments, act as insulators. These insulators can reduce the usage of the proximal *Trdv2-2* gene, thereby indirectly affecting *Tcra* rearrangement (2). *Tcrd* rearrangement in the earlier stage can eliminate the sequence between V_δ_ and DJ_δ_, bringing the V_α_ and J_α_ genes into close linear proximity. However, in intact alleles where V_δ_–DJ_δ_ rearrangement does not occur, the barrier to spatial proximity remains. Studies have demonstrated that CTCF and cohesin play a role in regulating *Tcra* rearrangement through long-range interactions (3,15). Within the *Tcra*–*Tcrd* locus, there are multiple CTCF/cohesin-binding elements (CBEs) (16), and several of these elements have been implicated in the regulation of *Tcra* rearrangement and the generation of TCR diversity (2,5).

The enhancer E_α_, located downstream of the *Tcra–Tcrd* locus, plays a crucial role in regulating *Tcra* rearrangement by activating the upstream promoter TEAp of the J_α_ gene array and establishing a recombination center in CD4^+^CD8^+^ double-positive (DP) thymocytes (17,18). E_α_ also regulates chromatin accessibility and germline transcription of the proximal V_α_ genes through long-range interactions (15). We recently reported that the EACBE, a CBE located immediately downstream of E_α_, augments the interactions between E_α_ and the proximal V_α_ genes, thereby promoting the primary rearrangement of *Tcra* (5). An intriguing question arises regarding whether E_α_/EACBE physically mediates the spatial proximity of V_α_ and J_α_ genes. This question encompasses two aspects: first, whether the proximal V_α_ region, the proximal J_α_ genes and E_α_/EACBE can interact simultaneously; second, whether the probability of interactions involving all three sites is higher than the probability of interactions between only two sites, thus exhibiting a synergic pattern. Analyzing the higher order chromatin structure of the locus holds the potential to provide insights into answering this question.

The 3C-HTGTS assay is a technique that combines chromosome conformation capture (3C) and high-throughput genome-wide translocation sequencing (HTGTS) (19–21). This method allows for the generation of high-resolution and reproducible interaction profiles between a specific genomic region of interest and the entire genome. We observed that 3C-HTGTS data contain extensive multiway chromatin interaction information, indicating the suitability of this method for analyzing higher order chromatin structure. We analyzed three-way contacts of the *Tcra* locus using 3C-HTGTS data to gain insights into the higher order chromatin structure of this region in DP thymocytes.

## MATERIALS AND METHODS

### Mice

Wild-type C57BL/6 mice were purchased from Guangdong Medical Animal Experimental Centre, while *Rag1*^−/−^ mice were kindly provided by Professor Wei Yang from the Department of Pathology, Southern Medical University, Guangzhou, China. EACBE^−/−^ mice were generated from strain C57BL/6 by Beijing Vitalstar Biotechnology (5). EACBE^−/−^ mice were bred with *Rag1*^−/−^ mice to generate EACBE^−/−^*Rag1*^−/−^ mice. All mice were housed in a specific pathogen-free facility managed by the Southern Medical University Division of Laboratory animal center. The handling of mice was conducted in accordance with protocols approved by the Southern Medical University Institutional Animal Care and Use Committee.

### Cell collection

To isolate DP thymocytes from *Rag1*^−/−^ mice, the mice were injected intraperitoneally with 150 μg of anti-CD3 antibody (2C11; Biolegend) at 3 weeks of age, and thymi were harvested and ground in MACS buffer 10 days after injection. Thymocytes were filtered through a 40 μm nylon mesh and then pelleted.

### 3C-HTGTS

3C-HTGTS libraries were prepared with DP thymocytes (8,20). In brief, 10 million cells were cross-linked with 1% formaldehyde at room temperature for 10 min and quenched with glycine (final concentration 0.125 M) on ice for 5 min. Cells were lysed and this was followed by adding 200 U of MboI to digest the chromatin overnight at 37°C with gentle shaking. MboI was inactivated by adding 10% sodium dodecylsulfate (SDS) to a final concentration of 1.5% and incubating at 37°C for 30 min. To reduce the SDS concentration, the solution was diluted with T4 ligase buffer containing 1% Triton X-100, followed by incubation at 37°C for 1 h. T4 ligase (New England Biolabs) was added and incubated overnight at 16°C. Cross-linking was reversed and samples were treated with proteinase K and RNase A prior to DNA extraction with 1:1 phenol–chloroform and precipitation with ethanol. 3C libraries were sonicated to 300–500 bp on a Qsonica Bioruptor Sonicator. Sonicated DNA was linearly amplified with a biotinylated primer (Supplementary Table S1) that anneals to sites of interest. Biotin-labeled single-stranded DNA products were enriched with streptavidin C1 beads (65001, Thermo Fisher Scientific), and followed by 3′-end ligation with the bridge adapter. The adapter-ligated products were amplified through nested polymerase chain reaction (PCR) using a nested primer and an adapter-complementary primer (Supplementary Table S1). Details of the primers used in this study are also listed in Supplementary Table S1. A final PCR for another 10–15 cycles of amplification with P5 and P7 primers was performed. After purification, libraries were sequenced on an Illumina NovaSeq 6000 platform to obtain 150 bp paired-end reads.

### 3C-HTGTS data processing for pairwise chromatin interactions

Paired-end Illumina sequencing fastq data were filtered by removing adapters and low-quality reads using fastp (v0.20.0) (22). Trimmed reads again were extracted from the sequence file after quality control with Cutadapt (v1.18). Paired-end reads containing a nested primer or adapter primer were merged manually using restriction enzyme recognition sequences into single reads with Pear (v0.9.6), then the first digested fragment behind the bait was obtained by splitting the single reads into fragments according to restriction enzyme recognition sequences. The remaining single-end reads were aligned to the enzyme-digested mm10 reference genome with Bowtie2 (v2.4.5, parameter: -p 8 –sensitive) (23), and the mouse genome sequence (mm10) was retrieved from the UCSC (http://hgdownload.cse.ucsc.edu/goldenPath/mm10/bigZips/chromFa.tar.gz); we extracted concordantly exact alignments using SAMtools (v1.9) (24). Self-ligation reads and off-target reads were filtered after mapping. For visualization, we converted the final bam files into bedGraph files using Bedtools (v2.29.2) (25). The signal peak bedGraph file was obtained by post-comparison filtering, signal statistics and standardization. We normalized bedGraph files using the CPM (counts per million in *cis*) normalization method and visualized them on the IGV genome browser. Differential pairwise interactions were identified by the R package R.4Cker (v1.0.0, k = 30) with the function nearBaitAnalysis called to define domains of interaction with the bait and DESeq2 (v1.34.0, *P* < 0.05) (26,27). Finally, we organized the results report and visualized it with the Bioconductor package ggplot2 (v3.3.6). The analysis of the correlation for experimental repetition used the R package corrplot (v.0.92).

### 4C data processing for pairwise chromatin interactions

Paired-end reads were obtained through quality filtering and adapter trimming using fastp (v0.20.0). The second restriction enzyme cut in 4C reads was removed. The first MboI fragment after the bait sequence was extracted and mapped to the enzyme-digested mm10 reference genome by Bowtie2 (v2.4.5, parameter: -p 8 –sensitive) in the form of single-end reads, and we extracted concordantly exact alignments using SAMtools (v1.9). We filtered self-ligation reads and off-target reads. For visualization, we converted the final bam files into bedGraph files using Bedtools (v2.29.2). Read numbers were counted and normalized by the CPM normalization method, and then files were visualized on the IGV genome browser. The analysis of the correlation for experimental repetition used the R package corrplot (v.0.92).

### 3C-HTGTS data processing for multiway chromatin interactions

Paired-end Illumina sequencing reads were filtered by removing adapters and low-quality reads using fastp (v0.20.0). Trimmed reads were extracted from the file after quality control with Cutadapt (v1.18). Paired-end reads containing a nested primer or adapter primer were merged at restriction enzyme cut sites into single reads with Pear (v0.9.6), then fragments with multiple MboI-cut sites were split and each fragment was aligned to the enzyme-digested mm10 reference genome by Bowtie2 (v2.4.5, parameter: -p 8 –sensitive). We extracted concordantly exact alignments using SAMtools (v1.9). We converted the final bam files into bed files using Bedtools (v2.29.2). Self-ligation and off-targeted fragments were filtered. Subsequently, we put all fragments retrieved from the same read according to the unique ID of each read on one line, then removed the continuous fragments. To create contact matrices, we extracted the first two digested fragments after the bait fragment, or a variety of combinations of three fragments were obtained by arranging all fragments from the same read. Raw contact matrices were generated at 3, 5 and 10 kb resolutions. For correction of raw contact matrices, these interaction counts were normalized for a total of 1 000 000 interactions at the same resolutions. Similar to a Hi-C matrix, coverage was represented in a two-dimensional matrix where each point represented the number of interactions found between two bins meaning a specific resolution. Visualization of three-way contact matrices was done with the R package GENOVA (v1.0.0) then differential analysis and visualization of local interactions from three-way interactions were obtained using the Bioconductor package DESeq2 (v1.34.0, *P* < 0.001) (28). Loops seen on the IGV genome browser were called using fixed-size bin resolutions from 3 to 10 kb. Briefly, interaction loops (contact frequencies ≥ 20) were identified by using raw contact frequencies. The distribution for the frequencies of unique reads containing the pairwise and multiway chromatin interactions was counted after aligning and filtering, and visualized with the Bioconductor package ggplot2 (v3.3.6).

### 4C data processing for multiway chromatin interactions

First, adapter sequences were trimmed with fastp (v0.20.0). We retained reads that were separated by MboI-cut sites. In other words, only fragments with two or more MboI sites were considered for analysis of multiway interactions. MboI-cut fragments after the bait sequence were split and individually aligned to the enzyme-digested mm10 reference genome by Bowtie2 (v2.4.5, parameter: -p 8 –sensitive). We extracted alignments using SAMtools (v1.9). The final bam files were converted into bed files using Bedtools (v2.29.2). We filtered the self-ligation and off-target fragments after mapping. Subsequently, we put all fragments retrieved from the same read on one line, then removed continuous fragments. To create contact matrices, we extracted the first two fragments after the bait or a variety of combinations of three fragments were obtained by arranging fragments from the same read. Raw contact matrices were generated at 3, 5 and 10 kb resolutions. For raw contact matrices correction, these interaction counts were normalized for a total of 1 000 000 interactions at the same resolutions. Like a Hi-C matrix, coverage was represented in a two-dimensional matrix where each point represented the number of interactions found between two bins meaning a specific resolution. A heatmap of three-way contact matrices was produced with the R package GENOVA (v1.0.0), and the distributions for the frequencies of unique reads containing the pairwise and multiway chromatin interactions were counted after aligning and filtering, and they were visualized with the Bioconductor package ggplot2 (v3.3.6).

### Bait–SOI analysis for three-way interactions for 3C-HTGTS

Similar to the method reported by Vermeulen *et al.* (29), we identified cooperative, random or competitive multiway contacts between the bait and two other sites of interest (SOIs), a SOI and any given third site, by performing an association analysis as follows. Briefly, if the interaction is a cooperative relationship between the bait, the SOI and the given third site, a subset of reads that contain both the bait and the SOI should frequently cover the given third site as well. To determine whether the third partner is cooperative, random or competitive, we compared the frequency of the third partner in the set of reads that contain both the bait and SOI (i.e. the positive set) with the frequency of the third partner in the set of reads that contain bait, but lack the SOI (i.e. the negative set). To account for technical and sampling variation that may occur, we randomly sample many reads from the negative set equal to the number of reads in the positive set, then filter one fragment from each sampled read in the negative set at random that can compensate for the SOI fragment present in all reads in the positive set. We repeated this procedure 1000 times to generate an average negative profile, and the mean and standard deviation (SD) were calculated correspondingly. The positive contact profile is then compared with the negative profile, and a *z*-score that is determined to evaluate the significance of cooperative or competitive contacts between the bait, the SOI and the third partner is calculated. The *z*-score closing to zero indicates a random contact frequency between the SOI and the third partner when bait is present, and a positive or negative *z*-score implies a cooperative or competitive contact between these three genomic regions.

Unique read counts of the third site in bait–SOI co-occurrence and in bait contacts without the SOI were respectively extracted from the positive set and negative set above, and box plots were generated with the Bioconductor package ggplot2 (v3.3.6).

## RESULTS

### 3C-HTGTS data contain sufficient information on multiway interactions

**Figure 1.**
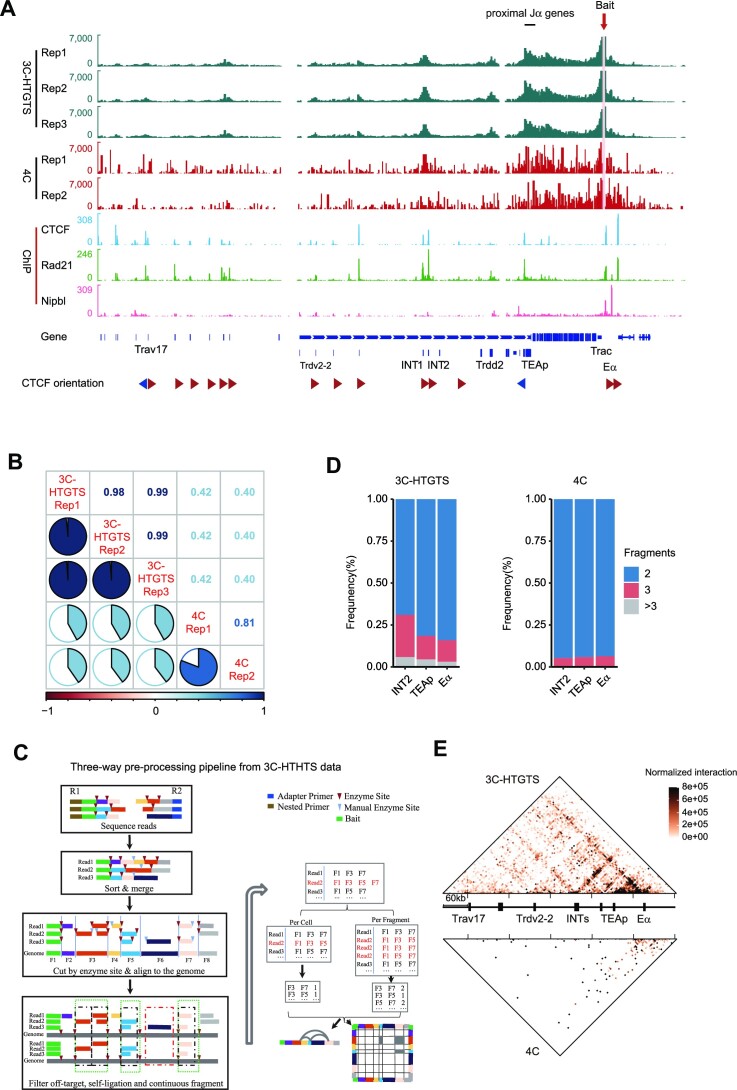
3C-HTGTS assay enables three-way contact analysis on the *Tcra*–*Tcrd* locus. (**A**) The 3C-HTGTS (teal) and 4C (red) tracks display the read density (top). The normalized signals represent pairwise chromatin interactions captured by the E_α_ bait in anti-CD3-induced DP thymocytes of *Rag1*^−/−^ or *Rag2*^−/−^ mice. The red arrow and the pink filled rectangle highlight the bait position. Representative tracks from two or three independent experiments are shown. Normalized CTCF, Rad21 and Nipbl ChIP-seq profiles in DP cells are displayed (below 4C tracks). The orientation of CTCF-binding motifs is indicated by arrowheads (forward orientation in red, reverse orientation in blue) at the bottom. (**B**) Comparison of the correlation of 3C-HTGTS and 4C experimental biological replicates. The data from the E_α_ bait are used here. (**C**) The pipeline for extracting three-way contacts from 3C-HTGTS data. (**D**) Stacked column plots showing the frequencies of reads containing multiway contacts from 3C-HTGTS and 4C data at the *Tcrα*–*Tcrd* locus. (**E**) Heatmap showing the comparison of three-way contacts captured by the E_α_ bait. The 3C-HTGTS data (top) have more three-way contacts than the 4C data (below). The heatmap represents one of three independent experiments. Resolution: 5 kb; coordinates (mm10): chr14:53740000–54300000.

To investigate the chromatin conformation of the *Tcra*–*Tcrd* locus in DP thymocytes, we performed a 3C-HTGTS assay using CD3-induced DP thymocytes of *Rag1*^−/−^ mice, with the baits of E_α_, the TEA promoter (TEAp), INT2 and a CBE upstream of the proximal V_α_ gene *Trav17* (*Trav17*–CBE2). The 3C-HTGTS assay generated higher resolution interaction profiles compared with the 4C data from the same baits (5,30) (Figure 1A; Supplementary Figure S1A). The biological replicates of the 3C-HTGTS profiles showed reproducibility (Figure 1B; Supplementary Figure S1B). The profiles clearly reveal strong interactions of E_α_ with the proximal J_α_ region (from *Traj61* to *Traj56*), as well as substantial interactions with INTs and several CBEs in the proximal V region (Figure 1A). Additionally, INTs and TEAp exhibited strong interactions with each other, and both of them displayed strong interactions with the D_δ_ gene *Trdd2* between them (Supplementary Figure S1A). INT2 and TEAp also have modest interactions with the CBEs in the proximal V region and with the downstream E_α_. These data indicate that the 3C-HTGTS technology provides a high-resolution and reproducible chromatin contact profile of a DNA sequence of interest in the locus.

To determine the suitability of the 3C-HTGTS assay for higher order chromatin structure analysis, we followed a pipeline to extract multiway contact information from the 3C-HTGTS data (Figure 1C; Supplementary Figure S1C). On average, 20% of 3C-HTGTS reads contain more than two fragments, whereas this proportion is only ∼7% in the 4C data (Figure 1D; Supplementary Figure S1D). We visualized the higher order structure in a contact matrix incorporating the three-way interactions of the E_α_ bait (Figure 1E; Supplementary Figure S1E). The contact matrix clearly demonstrates interactions of two elements from the E_α_ bait, and these higher order contacts exhibit high similarity across the three biological replicates. These data indicate that 3C-HTGTS can provide high-resolution and reproducible three-way contact profiles for higher order chromatin structures with a bait of interest.

### Three-way contact analysis exhibited a co-occurrence of the proximal V_α_ genes, the proximal J_α_ genes and E_α_ in DP thymocytes

**Figure 2.**
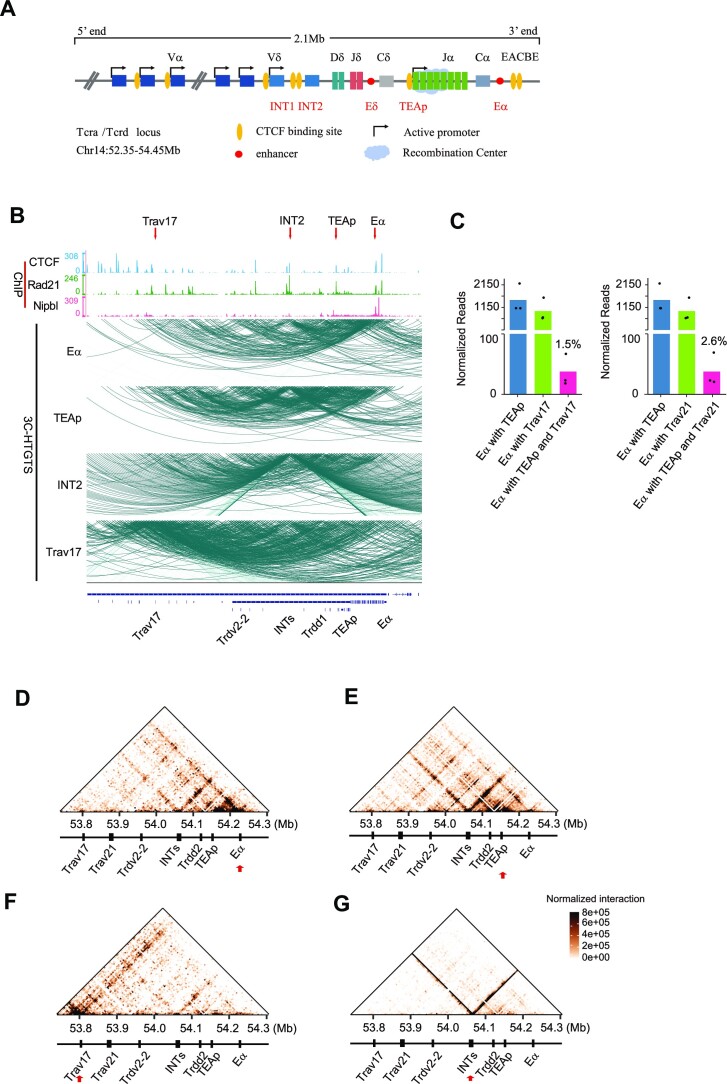
The proximal V_α_ genes, the proximal J_α_ genes and E_α_ form a chromatin hub in DP thymocytes. (**A**) Schematic diagram illustrating the linear structure of the *Tcrα–Tcrd* locus and its *cis*-regulatory elements. E_α_ and E_δ_ are enhancers of the *Tcra* and *Tcrd* genes, respectively. TEAp is the upstream promoter of the J_α_ gene array. EACBE is the CTCF-binding site located immediately downstream of E_α_. (**B**) ChIP-seq profiles of CTCF, Rad21 and Nipbl are depicted on the top. Genome browser tracks below display chromatin interactions of the second–third fragments in the 3′ portion of the *Tcra–Tcrd* locus from the bait of E_α_, TEAp, *Trav17* or INT2. The red arrows highlight the baits on the top. Strong interactions are observed between INTs and E_α_, as well as between the proximal V_α_ and J_α_ genes. Representative tracks for loops are shown from one of three independent experiments. Resolution: 5 kb; coordinates (mm10): chr14:53700000–54300000. (**C**) Comparison of read counts for E_α_–TEAp–*Trav17* (left) or E_α_–TEAp–*Trav21* (right) triplets and the proportions of these triplets relative to all pairwise interactions. Bar graphs represent the average normalized unique read counts in three replicates, with individual data points overlaid as dot plots. (D–G) Heatmaps showing three-way contacts of the 3′ portion of the *Tcra*–*Tcrd* locus from the bait of (**D**) E_α_, (**E**) TEAp, (**F**) *Trav17* or (**G**) INT2. The red arrow highlights the bait position. Each point represents the mean number of normalized unique interactions per restriction fragment in three replicates. Resolution: 5 kb; coordinates (mm10): chr14:53740000–54300000.

The *Tcra–Tcrd* locus has a complex organization due to the presence of two TCR genes, *Tcra* and *Tcrd*. The 5′ portion of the locus consists of the V gene region, which encompasses >100 V genes, some of which are shared by *Tcra* and *Tcrd*. Adjacent to the V gene region are the D_δ_, J_δ_ and C_δ_ segments of the *Tcrd* gene, with the enhancer E_δ_ between J_δ_ and C_δ_ (Figure 2A). At the 3′ end of the locus lies the J_α_ array, containing 59 J_α_ genes, along with the C_α_ segment. Further downstream is the enhancer E_α_. Multiple CBEs are present at the *Tcra–Tcrd* locus, particularly in the V_α_ gene region. This includes two CBEs, INT1 and INT2, situated between the V region and the *Tcrd* region, as well as a CBE at the TEA promoter, and the EACBE located downstream of E_α_. *Tcra* utilizes the ability of multiple rounds of rearrangement to generate a functional TCR capable of recognizing the major histocompatibility complex (MHC) (31). A recent work of the Krangel group showed that *Tcrd* rearrangement drives the usage of central and distal V_α_ segments in the *Tcra* primary rearrangement (32). The rearrangement primarily occurs between the proximal V_α_ genes and the proximal J_α_ genes on the *Tcrd*-intact allele. Therefore, this study focused on investigating interactions of the proximal V_α_ segments and the proximal J_α_ genes.

To understand the higher order chromatin structure in the 3′ portion of the *Tcra–Tcrd* locus in DP cells, we analyzed three-way contacts from the baits of E_α_, TEAp, INT2 and *Trav17–*CBE2. The 3C-HTGTS data obtained from these four baits contained enough multiway contacts for higher order chromatin structure analysis (Supplementary Figure S2A). In the baits of E_α_ and TEAp, the multiway contacts are primarily observed in the region spanning from *Trav17* to E_α_ (Figure 2B). From the E_α_ bait, strong interactions were observed between TEAp and the proximal V_α_ genes (Figure 2B, C). To better visualize the multiway interaction pattern, we generated heatmaps. The proximal J_α_ gene region showed significant interactions with INTs in the E_α_ bait (Figure 2D), probably mediated by the chromatin loop between TEAp–CBE and INT2 (Supplementary Figure S1A) (2). The proximal J_α_ region displayed substantial interactions with several CBEs in the proximal V region, particularly with the CBE upstream of the *Trav21* gene (Figure 2D). This result is consistent with the three-way contact heatmap in the TEAp bait, where E_α_ interacts strongly with INTs (Figure 2E). E_α_ also interacted with several V_α_ genes including *Trdv2-2, Trav21* and *Trav17* (Figure 2E). The multiway contact pattern was specific to the DP thymocytes, as it was not observed in liver cells (Supplementary Figure S2B, C). Collectively, these findings indicate the co-occurrence of contacts between the proximal V_α_ genes, the proximal J_α_ genes and E_α_ in DP thymocytes.

A notable feature observed in the *Trav17* heatmap is a broad chromatin contact stripe that extends from upstream of *Trav17* to E_α_ (Figure 2F). This stripe may be a result of cohesin extrusion from the CBEs located upstream of *Trav17* (Figure 1A). From the *Trav17* bait, TEAp displays substantial interactions with INTs (Figure 2F), supporting the co-occurrence of contacts between INTs, TEAp and the proximal V_α_ genes. We noticed a stripe extending from INTs to the proximal V region in the TEAp bait (Figure 2E). We then looked at the INT2 heatmap and saw a bidirectional stripe-like pattern of chromatin contacts. Most interactions formed two tight stripes, extending both upstream and downstream from the INTs (Figure 2G). This stripe structure appears to be constitutive, as it is also seen in liver cells (Supplementary Figure S2B). We previously reported a chromatin interaction stripe extending downstream from INT2 in a Hi-C heatmap of DP thymocytes (5). The stripe becomes more pronounced in the three-way contacts, and we can observe the stripe extending upstream as well. To confirm the presence of these stripes, we utilized the computational stripe detection tool, Stripenn (33). The statistical significance of the INT2 downstream stripe was detected in all four baits using Stripenn. Similarly, the INT2 upstream stripe was found to be statistically significant in the TEAp and INT2 baits (Supplementary Figure S2D). The result suggests that cohesin extrusion can occur both upstream and downstream from INTs.

### The proximal V_α_ genes are favored in the contacts involving the combination of the proximal J_α_ region and E_α_

The co-occurrence contacts of the proximal V_α_ region, the proximal J_α_ region and E_α_ prompted us to investigate whether E_α_ directly mediates the physical proximity of V_α_ and J_α_ genes. This implies that the interaction of E_α_ with either the J_α_ region or the V_α_ region favors the interaction with the other. Allahyar *et al.* present a method for analyzing specific three-way chromatin conformations that utilizes a second SOI to distinguish favored three-way contacts from random or disfavored contacts (34). To obtain statistically significant competing or cooperating sequences, this method compares the observed three-way co-occurrence frequency of each sequence with a given bait–SOI combination with its co-occurrence frequency in conformations of the bait without SOI in contacts (background) by calculating *z*-scores.

**Figure 3.**
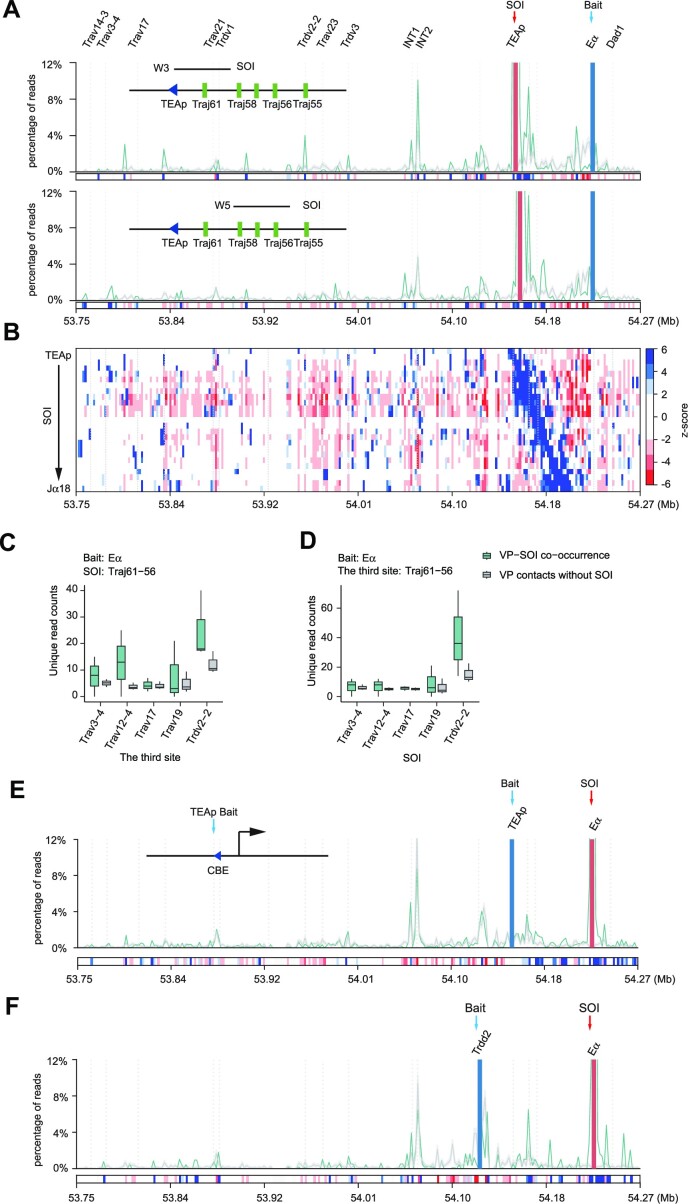
The TEAp–INT2 loop contributes to the three-way contacts of the E_α_, the proximal J_α_ and the proximal V_α_ genes. (**A**) Bait–SOI plot illustrating the co-occurrence contacts of sequences in the 3′ portion of the *Tcra*–*Tcrd* locus in the combination of the E_α_ bait and the SOI of the sequence containing *Traj61* (top) or *Traj56* (bottom). The green line represents the observed co-occurrence frequency of sequences across the locus, while the gray line represents the expected frequency (mean ± SD). The *z*-scores indicating significant enrichment or lacking a given site are shown in the bottom rectangles (dark blue for significant enrichment, dark red for significant absence of a given site). (**B**) Heatmap presenting the *z*-score landscape of sequences in the locus in co-occurrence contacts with SOI sliding windows (4 kb bin and 2 kb step) from TEAp to *Traj18* from the bait of E_α_. Rectangles in the heatmap represent the *z*-score values. (C and D) Box plots displaying the co-occurrence unique read counts in the combination of the E_α_ bait and (**C**) the SOI of the sequence containing *Traj61, Traj58, Traj57* and *Traj56* together, or (**D**) the sequences containing *Trav3-4, Trav12-4, Trav17, Trav19* or *Trdv2-2*, respectively. The third point represents the five V_α_ genes or the proximal J_α_ region. The green box plot represents the enrichment of the given third site in bait–SOI co-occurrence, and the gray box plot represents the enrichment of the given third site in bait contacts without the SOI. The data represent the mean ± SD. (E and F) Bait–SOI plots showing the co-occurrence contacts of sequences in the locus with the combination of the E_α_ SOI and (**E**) the TEAp bait or (**F**) the *Trdd2* bait.

First, we analyzed the co-occurrence frequency of any third sequence across the proximal *Tcra–Tcrd* region when the E_α_ bait interacts with the SOI *Traj61*, which is the first J_α_ gene commonly used in the primary rearrangement. Sequences immediately downstream of the SOI are favored in the three-way contact with the E_α_–*Traj61* combination, while sequences in the distal J_α_ region (except for one fragment) are disfavored (Figure 3A). The result is related to the role of E_α_ in regulating the TEA promoter through long-distance interactions (3). We observed that INT2 showed a high enrichment in the three-way contacts with the E_α_–*Traj61* combination (Figure 3A). The CBE in the TEA promoter and the INT2 are convergent, allowing them to form a tight chromatin loop (2), which may explain the strong co-occurrence of E_α_–*Traj61*–INT2. Additionally, several CBEs flanking *Trdv3, Trdv2-2, Trav18* and *Trav17* in the proximal V region are also favored in three-way contacts with the E_α_–*Traj61* combination (Figure 3A). This suggests that E_α_ physically promotes interactions between *Traj61* and the proximal V_α_ genes.

To understand the concurrency landscape of the proximal V_α_ and the proximal J_α_ region, we detected the enrichment of the locus using sliding 4 kb windows of SOIs. We observed that the preferred co-occurrence of the V region decreases rapidly as the sliding window leaves the proximal J_α_ region. Moreover, most sequences in the proximal V region are disfavored in combinations of E_α_ with SOIs downstream of the proximal J_α_ region (Figure 3A, B; Supplementary Figure S3). Overall, the J_α_ genes that exhibit concurrency with the combination of E_α_ and the V genes are primarily confined to a small region between TEAp and *Traj56*.

To gain a deeper understanding of three-way contacts involving E_α_, the proximal J_α_ genes and V_α_ genes, we utilized the *Traj61*–*Traj56* region as the SOI to extract the reads of five V_α_ genes as the third site (Figure 3C). *Trdv2-2, Trav3-4* and *Trav12-4* exhibit a higher number of reads in the co-occurrence of bait–SOI compared with bait contacts without the SOI. Similar results were seen when using the V genes as the SOI (Figure 3D). Since the bait we used did not reside in the restriction fragment containing E_α_, we designed a new primer in the E_α_ fragment for 3C-HTGTS (Supplementary Figure S4A–E). The 3C-HTGTS results using the E_α_ bait2 showed a high degree of similarity to the previous E_α_ bait. Although the co-occurrence of E_α_, the proximal J_α_ genes and V genes remains consistent, the signal is slightly lower (Supplementary Figure S4C–E). These findings indicate that the co-occurrence of the J_α_ region with the V genes and E_α_ is primarily limited to the proximal J_α_ genes, particularly *Traj61* and *Traj58*, which is consistent with the observation that *Tcra* primary rearrangement initiates at *Traj61* and *Traj58*. (5,14).

The characteristics of the interactions between the proximal J_α_ gene and the V_α_ genes become more evident when the V_α_ genes are used as the SOIs. A small proximal J_α_ region immediately downstream of TEAp, but not the TEAp itself, is favored in the three-way contacts when SOIs are *Trav3-4, Trav12-4, Trav17, Trav18, Trav19, Trav2-1* or *Trav2-2* (Supplementary Figure S5A, B). We speculated that while the TEAp–INT2 loop can promote interactions between the V_α_ genes and the proximal J_α_ gene, the interaction between TEAp and E_α_ disrupts the loop. To investigate further, we analyzed the co-occurrence frequency of the locus in the TEAp bait, where the bait is in the restriction fragment containing the TEAp–CBE. Our analysis revealed that INT2 is disfavored in the three-way contact with the TEAp–E_α_ (Figure 3E). Additionally, we examined three-way contacts from the *Trdd2* bait, which is located within the TEAp–INT2 loop. The analysis clearly demonstrates that INT2 is disfavored in the co-occurrence of the *Trdd2*–E_α_ combination (Figure 3F). Moreover, the proximal Vα region exhibits weak signals in the combination of E_α_ with TEAp or *Trdd2*. In conclusion, these results indicate that the TEAp–INT2 loop contributes to the three-way contacts of the E_α_, the proximal J_α_ and the proximal V_α_ genes.

### The TEAp–INT2 loop spatially separates the proximal V region from the *Tcrd* region within the loop

**Figure 4.**
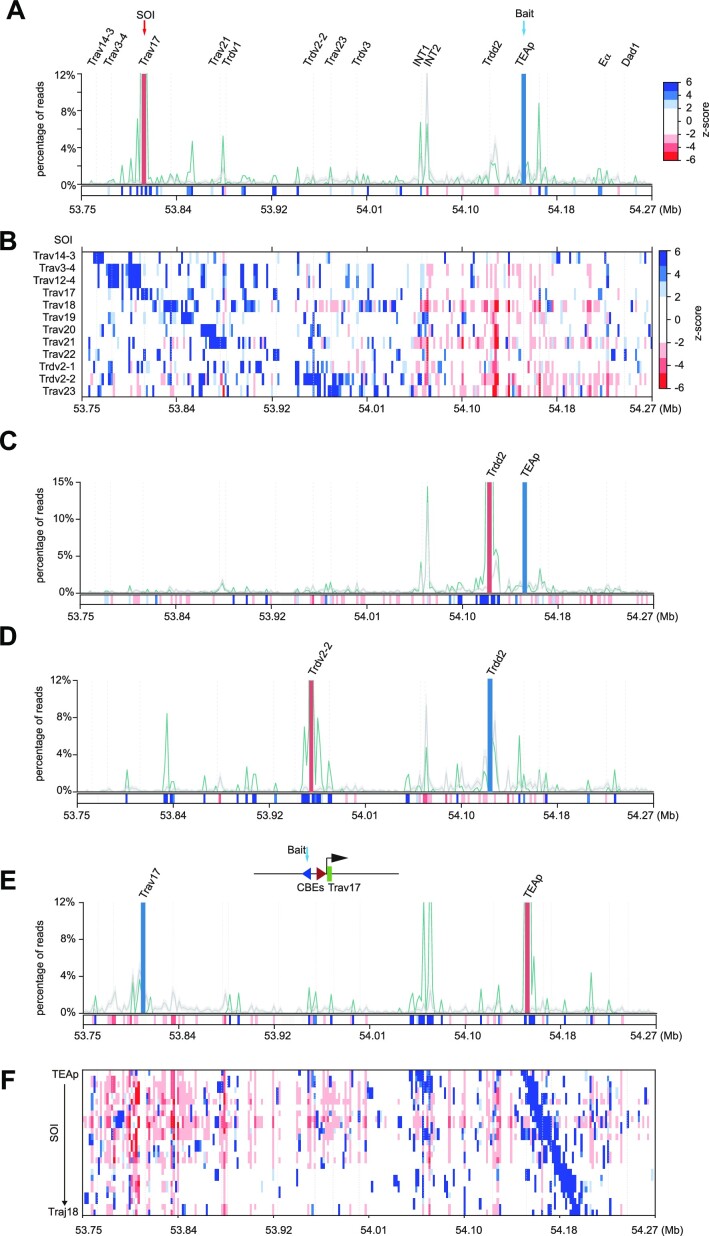
The *Tcrd* region is disfavored in the three-way contacts between the proximal J_α_ genes and the proximal V_α_ region. (**A**) Bait–SOI plot displaying the co-occurrence contacts of sequences in the locus with the combination of the TEAp bait and the *Trav17* SOI. The green line represents the observed co-occurrence frequency of sequences across the locus, while the gray line represents the expected frequency (mean ± SD). The *z*-scores indicating significant enrichment or lack of a given site are shown in the bottom rectangles (dark blue for significant enrichment, dark red for significant absence of a given site). (**B**) Heatmap showing the *z*-score landscape of sequences in the locus in co-occurrence contacts with the combination of the TEAp bait and the SOIs of V genes. Rectangles in the heatmap represent the value of the *z*-score. (C–E) Bait–SOI plots displaying co-occurrence contacts of sequences in the locus with the combinations of (**C**) the TEAp bait and the *Trdd2* SOI, (**D**) the *Trdd2* bait and the *Trdv2-2* SOI or (**E**) the *Trav17* bait and the TEAp SOI. (**F**) Heatmap showing the *z*-score landscape of sequences in the locus in co-occurrence contacts with the combination of the *Trav17* bait and the sliding windows (4 kb bin and 2 kb step) from TEAp to *Traj18*.

The Krangel group previously demonstrated that the TEAp–INT2 loop restricts the interaction of the *Trdv2-2* gene with D_δ_ genes, thereby limiting its rearrangement in the double-negative (DN) stage (2). To determine whether the TEAp–INT2 loop also restricts interactions with the V region in DP cells, we analyzed the enrichment of sites in the contacts of the V_α_–TEAp combination from the TEAp bait. We observed a preferential co-occurrence of multiple sites in the proximal V region in contacts involving the TEAp–*Trav17* combination (Figure 4A). This cooperative relationship in higher order chromatin conformation within the proximal V region is observed across all SOIs of V_α_ genes (Figure 4B; Supplementary Figure S6). However, we observed a rapid shift of the co-occurrence at INT2 (except for *Trav14-3* as the SOI) (Figure 4B). The disfavored region extends from INT2 to E_α_ (from INT2 to the proximal J_α_ for *Trav17* as the SOI), and there is a strongly disfavored region around *Trdd2* (Figure 4B; Supplementary Figure S6). Both INT1 and INT2 have strong signals but, interestingly, INT1 and INT2 do not behave in the same way: INT1, the left CBE, is favored in some SOI regions while INT2 is disfavored in almost all SOI regions (Figure 4A; Supplementary Figure S6). These results indicate that INT2 forms a strong chromatin loop with TEAp, impeding its interactions with the proximal V region.

Furthermore, the TEAp–INT2 loop also restricts the interaction of sequences within the loop with sequences outside the loop. When *Trdd2* was used as the SOI, it was evident that INT2 had a strong signal and was favored in the bait–SOI contacts (Figure 4C). However, there is no strong signal with the upstream V_α_ genes and downstream J_α_ genes, and lack of synergistic interactions. We also investigated the co-occurrence frequency of sequences within the locus using *Trdd2* as bait and Trdv2-2 as the SOI (Figure 4D). The results revealed that INT2 was disfavored in contacts with the bait–SOI combination, confirming that INT2 limits the interaction between *Trdd2* and *Trdv2-2*. This findings is consistent with the role of INT2 in *Tcrd* rearrangement in DN thymocytes (2).

We observed a chromatin stripe extending downstream from *Trav17* to E_α_ from the bait of the *Trav17* upstream CBE (*Trav17*–CBE2) (Figure 2F; Supplementary Figure S2D). To investigate this further, we examined the enrichment of sequences in the contacts of the combination of TEAp as the SOI from the *Trav17* bait. The most prominent feature was a strong preferential co-occurrence of two INTs when TEAp was used as the SOI (Figure 4E, F; Supplementary Figure S7). However, as the SOI slid downstream, the signals within INTs decreased and the preferred co-occurrence vanished. Interestingly, we noticed that INT2 was disfavored in three-way contacts involving TEAp–*Trav17* as the bait–SOI (Figure 4A), which contradicted the observation from the *Trav17*–CBE2 bait. We hypothesize that this discrepancy may be due to the different orientations of two CBEs upstream of *Trav17*. There are two CBEs upstream of *Trav17*, with the CBE closest to *Trav17* (*Trav17*–CBE1) oriented downstream, the same direction of most CBEs in the V region. On the other hand, *Trav17*–CBE2 is oriented upward, which is unique within the proximal V region. In the TEAp–*Trav17* bait–SOI, the SOI contains the *Trav17* gene body and CBE1 but not CBE2 (Figure 4A), while the bait is located at the DNA fragment containing *Trav17*–CBE2 in the *Trav17*–TEAp bait–SOI (Figure 4E). A recent study proposed a model in which cohesin-dependent loop anchors stack at the loop anchors, forming rosette-like structures (35). The co-occurrence of *Trav17*–INTs–TEAp contacts may reflect the formation of the chromatin hub by these CTCF-binding sites.

### The deletion of EACBE resulted in a reduced co-occurrence of V genes in the Eα–*Traj61* combination

**Figure 5.**
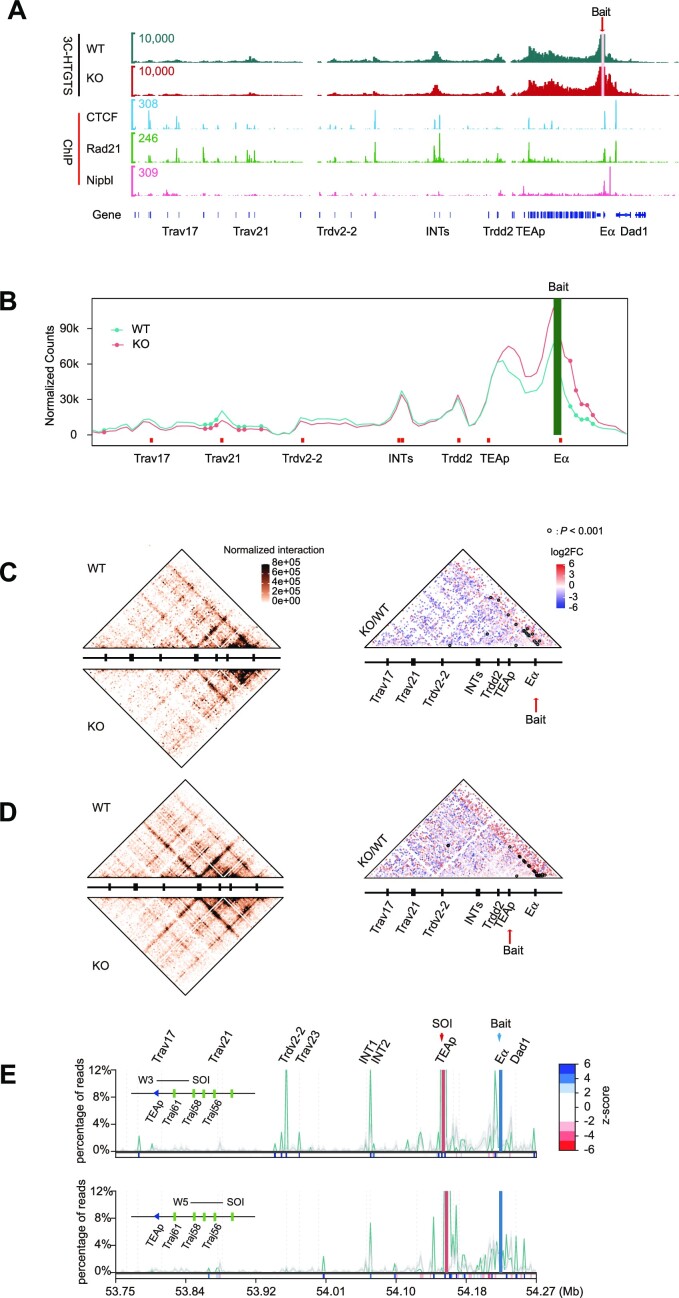
The deletion of EACBE resulted in a reduced co-occurrence of V genes in the Eα–*Traj61* combination. (**A**) 3C-HTGTS profiles of the E_α_ bait in anti-CD3-induced DP thymocytes of *Rag1*^−/−^ (WT) (teal) and EACBE^−/−^*Rag1*^−/−^ (KO) (red) mice. The EACBE deletion reduces the interactions between E_α_ and the proximal V_α_ region. Profiles represent the mean of normalized unique interactions for each restriction fragment in three replicates. Normalized CTCF, Rad21 and Nipbl ChIP-seq profiles in DP cells are displayed below. (**B**) Line plot displaying the difference of pairwise interactions between the WT and KO from the E_α_ bait using the 4C-ker program. The analysis was performed in three independent experimental replicates. Significant differential interactions (*P* < 0.05; statistics derived using DESeq2) are highlighted with filled circles. Interactions between E_α_ and the proximal V_α_ region significantly decrease in EACBE-deleted cells, while they increase in the region immediately downstream of E_α_. Gene positions are annotated by red-filled rectangles, and the green-filled bar highlights the bait position. (C and D) Heatmaps (left) and differential heatmaps (right) showing three-way chromatin interactions in (**C**) the E_α_ or (**D**) the TEAp bait in anti-CD3-induced DP thymocytes of *Rag1*^−/−^ (WT) and EACBE^−/−^*Rag1*^−/−^ (KO) mice. Statistically significantly different interactions are highlighted with black circles (*P* < 0.001). Interactions significantly decrease in the upstream region of EACBE and increase in the immediate downstream region. Resolution: 5 kb; coordinates (mm10): chr14:53740000–54300000. (**E**) Bait–SOI plot displaying three-way contacts in the DP thymocytes of EACBE^−/−^*Rag1*^−/−^ mice. Bait (blue rectangle), E_α_; SOI (red rectangle), *Traj61* (top) and *Traj56* (bottom). The green line represents the observed co-occurrence frequency of sequences, while the gray line represents the expected frequency (mean ± SD). The *z*-scores indicating significant enrichment or lack of a given site are shown in the bottom rectangles (dark blue for significant enrichment, dark red for significant absence).

Our previous study showed that EACBE deletion reduced the interaction between E_α_ and proximal V_α_ genes, thus impairing *Tcra* rearrangement (5). To investigate the role of EACBE in the higher order chromatin structures of the *Tcra*–*Tcrd* locus, we performed 3C-HTGTS assays with DP thymocytes of EACBE^−/−^*Rag1*^−/−^ mice. We observed increased interactions of E_α_ with flanking DNA sequences, especially the downstream sequences (Figure 5A, B). Eα exhibited substantial interactions with the region containing *Trav21* and *Trdv1* genes, which was reduced in EACBE^−/−^ mice (Figure 5A, B). The results are consistent with our previous observation using the 4C assay (5). Multiway contacts with the E_α_ bait demonstrated an increase in contacts between EACBE downstream sequences and the upstream region in EACBE-deleted cells, indicating its insulator function (Figure 5C). Furthermore, we observed a global reduction in three-way contacts in the TEAp upstream region in EACBE^–/–^ DP thymocytes, which may be attributed to the reduced interactions of E_α_ with the proximal V region.

To understand how EACBE affects the higher order chromatin conformation of the proximal V region, we performed a 3C-HTGTS assay with TEAp and *Trav17*–CBE2 baits. We observed a substantial reduction of contacts between TEAp and the upstream region, particularly INTs and the *Trav21* gene, in EACBE-deleted DP thymocytes (Supplementary Figure S8A, B). Consistent with this, *Trav17* exhibited a reduction of contacts with the downstream region from *Trav21* to E_α_ (Supplementary Figure S8A, B). Multiway contact analysis demonstrated that EACBE deletion led to a reduction in the INTs stripe towards the upstream region from the TEAp bait, and the reduction of INTs–*Trav21* interactions was statistically significant (Figure 5D). The multiway contact data from the *Trav17* bait also showed reduced interactions with the downstream region (Supplementary Figure S8C). The bait–SOI analysis revealed that INT2 was favored in the three-way contacts with the E_α_–*Traj61* combination, similar to the WT (Figure 5E). Unlike multiple V_α_ genes with preferential co-occurrence contacts in the E_α_–*Traj61* combination in WT thymocytes, only *Trdv2-2* and *Trav23* genes were favored in three-way contacts with the E_α_–*Traj61* combination (Figure 5E). This may be due to reduced interactions between E_α_ and V genes. In summary, although EACBE promotes interactions of E_α_ with the V genes, its deletion did not affect the co-occurrence of the E_α_–*Traj61–*INT2, indicating the role of the TEAp–INT2 loop in the higher order structure of the *Tcra* locus.

## DISCUSSION

Understanding higher order chromatin structures is crucial for comprehending the mechanisms of *cis*-regulatory elements in gene expression regulation, genome replication, V(D)J recombination and other genome metabolism processes. Several multiway contact techniques have been developed for investigating higher order chromatin structures (36–40). To obtain sufficient multiway contact information for the analysis of higher order chromatin structures, some studies have utilized long-read sequencing technology, such as MC-4C technology (34). More recently, the Pore-C technique, which combines Hi-C and nanopore sequencing technology, has been developed for the genome-wide detection of higher order chromatin structures (41,42). Here we found that a 3C-HTGTS assay on the Illumina sequencing platform can provide 15–30% of the multiway information of the locus of interest, offering a convenient solution for studying higher order chromatin structures at the locus of interest.

Using 3C-HTGTS, we examined the higher order structure of the *Tcra–Tcrd* locus in CD4^+^CD8^+^ DP thymocytes, where *Tcra* undergoes active rearrangement. The *Tcrd* gene is located inside the *Tcra* gene, separating V_α_ genes and J_α_ genes. It has two effects on the *Trca* rearrangement. (i) The V_δ_–DJ_δ_ rearrangement of *Tcrd* in the DN stage can delete a partial sequence between the V region and the J_α_ region, including INTs. The deletion may promote the V_α_–J_α_ rearrangement and increase the diversity of *Tcra* (32). (ii) On alleles where *Tcrd* does not undergo V_δ_–DJ_δ_ rearrangement, it can act as an obstacle and impede *Tcra* rearrangement. E_α_ is an essential enhancer downstream of *Tcra* and is required for chromatin accessibility in the J_α_ gene region. The Krangel group reported that E_α_ regulates the chromatin accessibility of the proximal V region through long-distance interactions (15). Our study on EACBE also revealed its role in mediating interactions of E_α_ with the proximal V region (5). Here, we provide physical evidence of the co-occurrence contacts of E_α_, the V_α_ genes and the proximal J_α_ genes, particularly *Traj61* and *Traj58*. It has been reported that deletion of E_α_ results in a decrease in the interactions between TEAp and *Trav21*, TEAp and *Trdv2-2*, and *Trav21* and *Trdv2-2* (15). While the reduction in chromatin activity due to the loss of the enhancer may contribute to this decrease in interactions, it is also possible that the disruption of synergistic physical three-way contacts partially explains this reduction. Interestingly, only the J_α_ genes immediately following TEA exhibit preferential co-occurrence contacts, consistent with the observation that the primary rearrangement of *Tcra* starts from the proximal J_α_ gene (14).

Chen *et al.* reported that INT2 restricts the usage of *Trdv2-2* in *Tcrd* rearrangement in DN thymocytes by forming a chromatin loop between INT2 and the CBE in the TEA promoter (2). Normally, V_δ_–DJ_δ_ rearrangement deletes INTs, bringing the V gene region in close proximity to the J_α_ gene region linearly. However, on the allele that does not undergo V_δ_–DJ_δ_ rearrangement, how do INTs affect the interaction of V_α_ and J_α_ genes? Our observations reveal that a strong chromatin loop still exists between the TEA promoter and INT2 in DP thymocytes. The TEAp–INT2 loop plays a dual role in the higher order structure of the *Tcrd*–*Tcrd* locus in DP thymocytes: (i) it brings the proximal V_α_ genes closer to the recombination center formed by the proximal J_α_ genes and E_α_; and (ii) the loop restricts the sequences in the loop interacting with sequences outside of the loop, potentially repressing *Tcrd* rearrangement in DP stages.

Here we developed a pipeline for analyzing multiway contacts in 3C-HTGTS data, which offers a cost-effective and straightforward approach for investigating the higher order chromatin structure of a specific genomic locus. By employing this pipeline, we conducted an analysis of the higher order chromatin conformation in the 3′ portion of the *Tcra–Tcrd* locus in DP thymocytes. Our findings provide compelling evidence for the co-occurrence contacts of E_α_, the V_α_ genes and the proximal J_α_ genes, establishing a paradigm for the study of higher order chromatin structure.

## Supplementary Material

gkad641_Supplemental_File

## Data Availability

For results generated from public sequencing, data information has been provided in the figure legends. In addition to the emphasized public data, other sequencing data generated in this study have been deposited at the NCBI GEO under accession number GSE214918. All the accession numbers of sequencing data are summarized in Supplementary Table S2. The code used in this manuscript is available at FigShare https://figshare.com/s/aee7de7801c00b1df137 doi:10.6084/m9.figshare.21666113.
