# Supplementary material for: Three-way contact analysis characterizes the higher order organization of the Tcra locus
Source: Nucleic Acids Res. 2023 Aug 3;51(17):8987–9000. doi: 10.1093/nar/gkad641 (PMC10516640; doi:10.1093/nar/gkad641)
Supplement: gkad641_Supplemental_File [file gkad641_supplemental_file.pdf]

Table S1 Primer sequences for 3C HTGTS assay

Bait	Biotin Primer	Nested Prime	Adapter Prime
E_α	5'-cgtcgaagagtctctgcctg	5'-cttgttctgattggatggcga	5'-gactatagggcacgcgtgg
$E_\alpha 2$	5'-ctgctgacatgggcaaacag	5'-catcctctggaaagaggagtt	5'-gactatagggcacgcgtgg
INT2	5'-accacagaaacccaacagtg	5'-tggctttctctccttggtca	5'-gactatagggcacgcgtgg
TEAp	5'-tagcccatctccataaacaggt	5'-ggtcaaagaaggaaaacactctc	5'-gactatagggcacgcgtgg
Trdd2	5'-gcctgaactaactgccaacc	5'-tacaaagcccagggaaggtt	5'-gactatagggcacgcgtgg
Trav17	5'-tcccacagcaggaaaacaga	5'-aagctacaccgagacaagga	5'-gactatagggcacgcgtgg

Table S2 NGS data used in this paper

Data	Source	Accession
4C (*Rag2*$^{-/-}$ DP) E_α rep1	(Hao Zhao et al., 2020)	GEO: GSM4307209
4C (*Rag2*$^{-/-}$ DP) E_α rep2	(Hao Zhao et al., 2020)	GEO: GSM4307210
4C (*Rag2*$^{-/-}$ DP) INT2 rep1	(Hao Zhao et al., 2020)	GEO: GSM4307213
4C (Rag2$^{-/-}$ DP) INT2 rep2	(Hao Zhao et al., 2020)	GEO: GSM4307214
4C (Rag2$^{-/-}$ DP) TEAp rep1	(Hao Zhao et al., 2020)	GEO: GSM4307217
4C (Rag2$^{-/-}$ DP) TEAp rep2	(Hao Zhao et al., 2020)	GEO: GSM4307218
3C-HTGTS (*Rag1*$^{-/-}$ DP) E_α rep1	This paper	GEO: GSE214918
3C-HTGTS (*Rag1*$^{-/-}$ DP) E_α rep2	This paper	GEO: GSE214918
3C-HTGTS (*Rag1*$^{-/-}$ DP) E_α rep3	This paper	GEO: GSE214918
3C-HTGTS (Rag1$^{-/-}$ DP) E_α bait2	This paper	GEO: GSE214918
3C-HTGTS (*Rag1*$^{-/-}$ DP) INT2 rep1	This paper	GEO: GSE214918
3C-HTGTS (*Rag1*$^{-/-}$ DP) INT2 rep2	This paper	GEO: GSE214918
3C-HTGTS (*Rag1*$^{-/-}$ DP) INT2 rep3	This paper	GEO: GSE214918
3C-HTGTS (*Rag1*$^{-/-}$ DP) TEAp rep1	This paper	GEO: GSE214918
3C-HTGTS (*Rag1*$^{-/-}$ DP) TEAp rep2	This paper	GEO: GSE214918
3C-HTGTS (*Rag1*$^{-/-}$ DP) TEAp rep3	This paper	GEO: GSE214918
3C-HTGTS (*Rag1*$^{-/-}$ DP) *Trdd2* rep1	This paper	GEO: GSE214918
3C-HTGTS (*Rag1*$^{-/-}$ DP) *Trdd2* rep2	This paper	GEO: GSE214918
3C-HTGTS (*Rag1*$^{-/-}$ DP) *Trdd2* rep3	This paper	GEO: GSE214918
3C-HTGTS (*Rag1*$^{-/-}$ DP) *Trav17* rep1	This paper	GEO: GSE214918
3C-HTGTS (*Rag1*$^{-/-}$ DP) *Trav17* rep2	This paper	GEO: GSE214918
3C-HTGTS (*Rag1*$^{-/-}$ DP) *Trav17* rep3	This paper	GEO: GSE214918
3C-HTGTS (EACBE$^{-/-}$ *Rag1*$^{-/-}$ DP) E_α rep1	This paper	GEO: GSE214918
3C-HTGTS (EACBE$^{-/-}$ *Rag1*$^{-/-}$ DP) E_α rep2	This paper	GEO: GSE214918
3C-HTGTS (EACBE$^{-/-}$ *Rag1*$^{-/-}$ DP) E_α rep3	This paper	GEO: GSE214918
3C-HTGTS (EACBE$^{-/-}$ *Rag1*$^{-/-}$ DP) TEAp rep1	This paper	GEO: GSE214918
3C-HTGTS (EACBE$^{-/-}$ *Rag1*$^{-/-}$ DP) TEAp rep2	This paper	GEO: GSE214918
3C-HTGTS (EACBE$^{-/-}$ *Rag1*$^{-/-}$ DP) TEAp rep3	This paper	GEO: GSE214918
3C-HTGTS (EACBE$^{-/-}$ *Rag1*$^{-/-}$ DP) *Trav17* rep1	This paper	GEO: GSE214918
3C-HTGTS (EACBE$^{-/-}$ *Rag1*$^{-/-}$ DP) *Trav17* rep2	This paper	GEO: GSE214918
3C-HTGTS (EACBE$^{-/-}$ *Rag1*$^{-/-}$ DP) *Trav17* rep3	This paper	GEO: GSE214918
3C-HTGTS (*Rag1*$^{-/-}$ Liver) E_α rep1	This paper	GEO: GSE214918

3C-HTGTS (*Rag1*$^{-/-}$ Liver) E_α rep2	This paper	GEO: GSE214918
3C-HTGTS (*Rag1*$^{-/-}$ Liver) INT2 rep1	This paper	GEO: GSE214918
3C-HTGTS (*Rag1*$^{-/-}$ Liver) INT2 rep2	This paper	GEO: GSE214918
3C-HTGTS (*Rag1*$^{-/-}$ Liver) TEAp rep1	This paper	GEO: GSE214918
3C-HTGTS (*Rag1*$^{-/-}$ Liver) TEAp rep2	This paper	GEO: GSE214918
3C-HTGTS (*Rag1*$^{-/-}$ Liver) *Trav17* rep1	This paper	GEO: GSE214918
3C-HTGTS (*Rag1*$^{-/-}$ Liver) *Trav17* rep2	This paper	GEO: GSE214918
Rad21 ChIP-seq (DP)	(Loguercio S et al., 2018)	GEO: GSM2973692
Nipbl ChIP-seq (DP)	(Merkenschlager M et al., 2013)	GEO: GSM1184315
CTCF ChIP-seq (DP)	(Torkamani A, et al., 2012)	GEO: GSM1023418

Figure S1 Related to figure 1.

a) The 3C-HTGTS (teal) and 4C (red) tracks display read density. Normalized signals represent pairwise interactions captured with the baits of *Trav17*, INT2, or TEAp in anti-CD3-induced DP thymocytes of *Rag1*$^{-/-}$ or *Rag2*$^{-/-}$ mice. The pink-filled rectangles highlight the bait position. Representative tracks are from two or three independent experiments. Normalized CTCF, Rad21 and Nipbl ChIP-seq profiles in DP cells are shown below the 4C tracks.

b) Correlation of 3C-HTGTS and 4C experimental replicates. INT2 and TEAp are used as baits, respectively.

c) Overview of the experimental procedure and acquisition of 3C-HTGTS libraries, including crosslinking, digestion, ligation, crosslink reversal, and sonication. DNA fragments containing the bait sequence is linearly amplified using biotin-labeled primers. Following capture with streptavidin beads, DNA fragments are ligated with adaptors for library amplification. Libraries are sequenced on an Illumina sequencing platform.

d) Boxplot showing frequencies of *cis*-triplet fragments in 3C-HTGTS data.

e) Heatmaps showing three-way contacts in the second and third replicates in the E_α bait. Annotation is shown below the heatmaps. Resolution: 5kb; Coordinates (mm10): chr14:53740000-54300000.

Figure S2 Related to figure 2

a) Bar graph showing frequencies of reads containing multi-way contacts in anti-CD3-induced DP thymocytes of Rag1$^{-/-}$ (WT) and EACBE$^{-/-}$ *Rag1*$^{-/-}$ (KO) mice.

b) Heatmaps showing three-way contacts in the baits of E_{α}, TEAp, *Trav17*, and INT2 in liver cells of the *Rag1*$^{-/-}$ mice. The red arrows highlight the bait positions. Points represent the mean of normalized unique interactions for each restriction fragment in two replicates. Annotation and coordinates are shown below the heatmaps. Resolution: 5kb; Coordinates (mm10): chr14:53740000-54300000.

c) Subtraction heatmaps showing the difference in three-way contact between DP thymocytes and liver cells from the four baits. Points represent the mean of normalized unique interactions per restriction fragment in three (DP cells) or two (liver cells) experimental replicates.

d) Representative stripes detected using Stripenn are delineated on three-way contact matrices of 3C-HTGTS in the four baits, respectively. Stripes are marked with teal-lined rectangles, and the *p*-value for each stripe is shown.

Figure S3 Related to Figure 3a and 3b

Bait-SOI plots illustrating the co-occurrence contacts of sequences in the locus with the combination of the E_α bait and the sliding windows (4kb bin and 2kb step) of SOIs from TEAp to *Traj18*. The green line represents the observed co-occurrence frequencies of sequences, while the gray line represents the expected co-occurrence frequencies (mean ± s.d.) across the locus. The z-scores indicating the significance of enrichment or lack of a given site are displayed in the bottom rectangles. Dark blue color indicates significant enrichment, while dark red color indicates a significant lack of a given site.

Figure S4 The E_{α} promotes the proximity of the proximal J_{α} genes to the V_{α} region in DP thymocytes.

a) 3C-HTGTS signal counts (teal) of the 3' portion of the *Tcra*-*Tcrd* locus, from the E_{α} -containing fragment (bait2). The normalized CTCF ChIP-seq profile (blue) in DP cells is shown below the 3C-HTGTS track.

b) Heatmap showing three-way contacts of the 3' portion of the *Tcra*-*Tcrd* locus. Resolution: 5kb; Coordinates (mm10): chr14:53740000-54300000

c) Bait-SOI plot displaying the co-occurrence contacts of sequences in the locus with the combination of the E_{α} bait and the SOI of the *Traj61*-to-*Traj56* region.

d) and **e**) Bar graphs displaying the co-occurrence unique read counts in the combination of the E_{α} bait2 and the SOI of (**d**) the sequence of the proximal J_{α} region or (**e**) the sequences containing *Trav3-4*, *Trav12-4*, *Trav17*, *Trav19* , or *Trdv2-2*, respectively. The third points represent the five V_{α} genes or the proximal J_{α} region. The green bars represent the enrichment of the given third site in bait-SOI co-occurrence, and the gray bars represent the enrichment of the given third site in bait contacts without SOI. The data represent mean ±s.d.

Figure S5 The proximal J_α region is disfavored in contacts with the combination of the E_α bait and SOIs of V genes.

a) Bait-SOI plots displaying the co-occurrence contacts of sequences in the locus with the combination of the E_α bait and the SOIs of V_α genes. The green line represents the observed co-occurrence frequency of sequences, and the gray line represents the expected (mean ± s.d.) co-occurrence frequency of sequences across the locus.

b) Heatmap showing the z-score landscape of sequences in the locus in co-occurrence contacts with the combination of the E_α bait and the SOIs of V genes. Rectangles in the heatmap represent the value of z-score.

Figure S6 Related to Figure 4b

Bait-SOI plots displaying co-occurrence contacts of sequences in the locus with the combination of the TEAp bait and the SOIs of the V genes. The green line represents the observed co-occurrence frequency of sequences, and the gray line the expected (mean ± s.d.) co-occurrence frequency of sequences. The z-scores are shown in the bottom rectangles, with dark blue indicating significant enrichment and dark red indicating significant lack of a given site.

Figure S7 Related to Figure 4f

Bait-SOI plot displaying co-occurrence contacts of sequences in the locus with the combination of the *Trav17* bait and the SOI sliding windows (4kb bin and 2kb step) from TEAp to Traj18. The green line represents the observed co-occurrence frequency of sequences, and the gray line represents the expected (mean ± s.d.) co-occurrence frequency of sequences.

Figure S8 Related to figure 5

a) 3C-HTGTS pair-wise chromatin interactions profiles of the 3' portion of the *Tcrα-Tcrd* locus in anti-CD3-induced DP thymocytes of *Rag1*$^{-/-}$ (WT) (teal) and EACBE$^{-/-}$ *Rag1*$^{-/-}$ (KO) (red) mice. Bait: *Trav17* or TEAp. Normalized CTCF, Rad21 and Nipbl ChIP-seq profiles in DP cells are displayed below.

b) Line plot displaying significant differences in pairwise chromatin interactions. The 4C-ker program was used for statistical analysis. Bait: *Trav17* (top) or TEAp (bottom). The analysis was performed with three independent experimental replicates. Filled circles in the line plot highlight interactions with statistically significant differences ($P < 0.05$).

c) Heatmap (Left) and subtraction heatmap (right) showing three-way contacts in the *Trav17* bait in anti-CD3-induced DP thymocytes of *Rag1*$^{-/-}$ (WT) and EACBE$^{-/-}$ *Rag1*$^{-/-}$ (KO) mice. Statistically significant different interactions are highlighted with black circles ($P < 0.001$). Gene annotation is shown below the heatmaps. Resolution: 5kb; Coordinates(mm10): chr14:53740000-54300000.

Figure S1 Related to figure 1.

a) The 3C-HTGTS (teal) and 4C (red) tracks display read density. Normalized signals represent pairwise interactions captured with the baits of *Trav17*, INT2, or TEAp in anti-CD3-induced DP thymocytes of *Rag1*$^{-/-}$ or *Rag2*$^{-/-}$ mice. The pink-filled rectangles highlight the bait position. Representative tracks are from two or three independent experiments. Normalized CTCF, Rad21 and Nipbl ChIP-seq profiles in DP cells are shown below the 4C tracks.

b) Correlation of 3C-HTGTS and 4C experimental replicates. INT2 and TEAp are used as baits, respectively.

c) Overview of the experimental procedure and acquisition of 3C-HTGTS libraries, including crosslinking, digestion, ligation, crosslink reversal, and sonication. DNA fragments containing the bait sequence is linearly amplified using biotin-labeled primers. Following capture with streptavidin beads, DNA fragments are ligated with adaptors for library amplification. Libraries are sequenced on an Illumina sequencing platform.

d) Boxplot showing frequencies of *cis*-triplet fragments in 3C-HTGTS data.

e) Heatmaps showing three-way contacts in the second and third replicates in the E_α bait. Annotation is shown below the heatmaps. Resolution: 5kb; Coordinates (mm10): chr14:53740000-54300000.

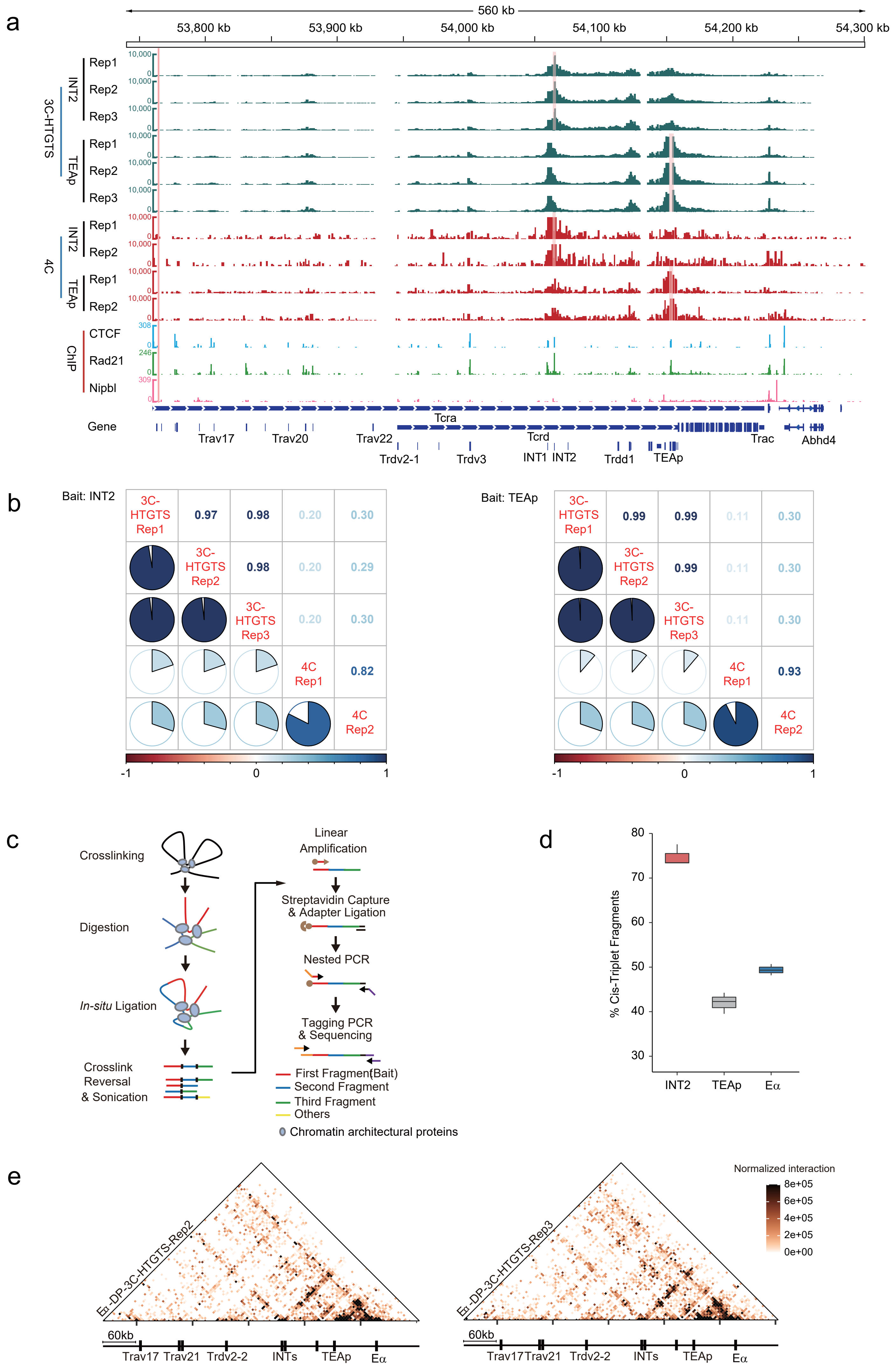
a
560 kb
53,800 kb
53,900 kb
54,000 kb
54,100 kb
54,200 kb
54,300 kb
3C-HTGTS
INT2
TEAp
Rep1
Rep2
Rep3
4C
ChIP
CTCF
Rad21
Nipbl
Gene
Tcra
Tcrd
Trav17
Trav20
Trav22
Trdv2-1
Trdv3
INT1 INT2
Trdd1
TEAp
Trac
Abhd4
b
Bait: INT2
Bait: TEAp
3C-HTGTS Rep1
3C-HTGTS Rep2
3C-HTGTS Rep3
4C Rep1
4C Rep2
0.97
0.98
0.20
0.30
0.29
0.82
0.99
0.11
0.93
-1
0
1
c
Crosslinking
Digestion
In-situ Ligation
Crosslink Reversal & Sonication
Linear Amplification
Streptavidin Capture & Adapter Ligation
Nested PCR
Tagging PCR & Sequencing
First Fragment(Bait)
Second Fragment
Third Fragment
Others
Chromatin architectural proteins
d
% Cis-Triplet Fragments
80
70
60
50
40
30
INT2
TEAp
Eα
e
Eα-DP-3C-HTGTS-Rep2
Eα-DP-3C-HTGTS-Rep3
Normalized interaction
8e+05
6e+05
4e+05
2e+05
0e+00
60kb
Trav17
Trav21
Trdv2-2
INTs
TEAp
Eα

Figure S2 Related to figure 2

a) Bar graph showing frequencies of reads containing multi-way contacts in anti-CD3-induced DP thymocytes of Rag1$^{-/-}$ (WT) and EACBE$^{-/-}$ *Rag1*$^{-/-}$ (KO) mice.

b) Heatmaps showing three-way contacts in the baits of E_{α}, TEAp, *Trav17*, and INT2 in liver cells of the *Rag1*$^{-/-}$ mice. The red arrows highlight the bait positions. Points represent the mean of normalized unique interactions for each restriction fragment in two replicates. Annotation and coordinates are shown below the heatmaps. Resolution: 5kb; Coordinates (mm10): chr14:53740000-54300000.

c) Subtraction heatmaps showing the difference in three-way contact between DP thymocytes and liver cells from the four baits. Points represent the mean of normalized unique interactions per restriction fragment in three (DP cells) or two (liver cells) experimental replicates.

d) Representative stripes detected using Stripenn are delineated on three-way contact matrices of 3C-HTGTS in the four baits, respectively. Stripes are marked with teal-lined rectangles, and the *p*-value for each stripe is shown.

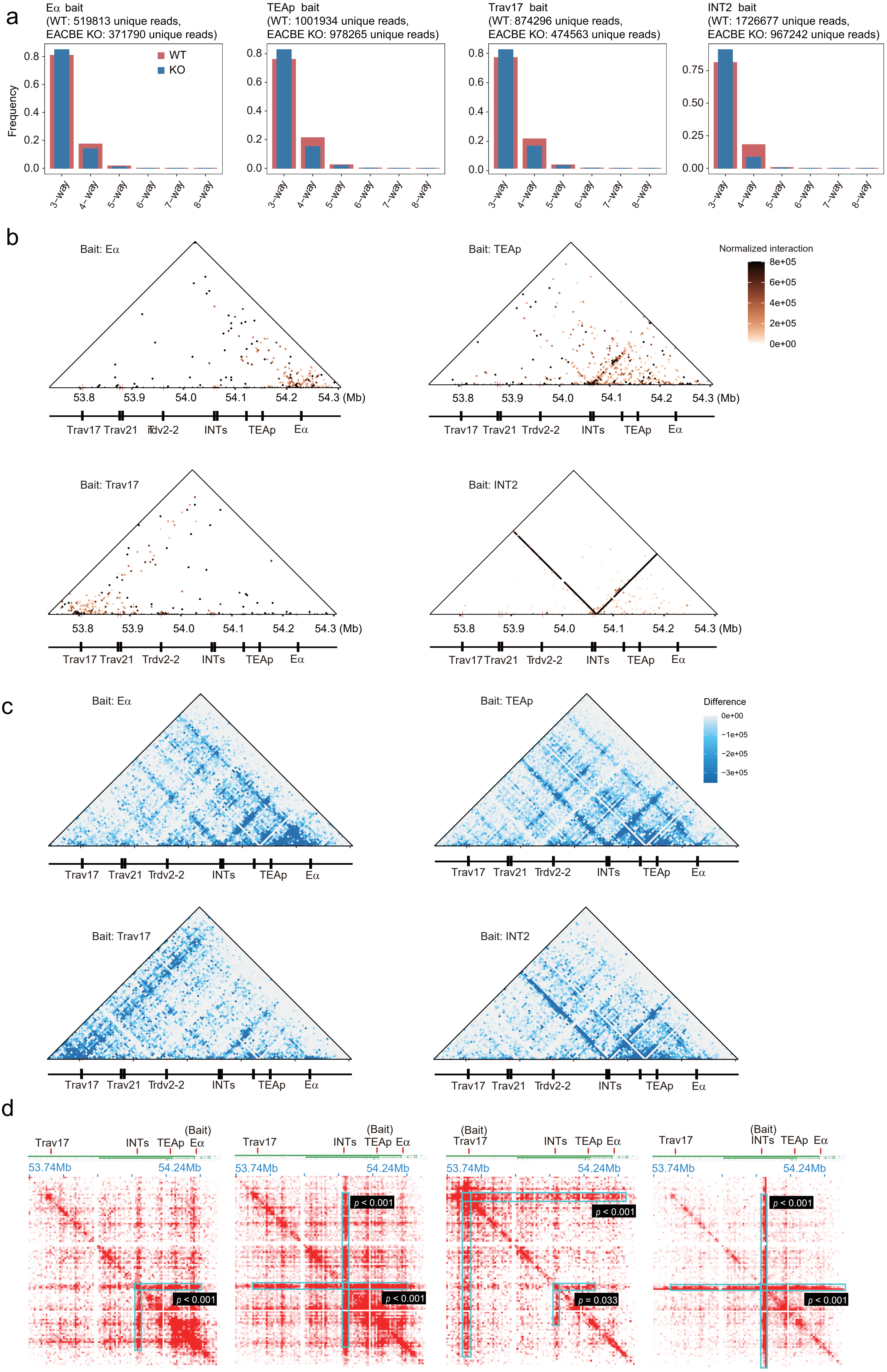
a
Eα bait
(WT: 519813 unique reads,
EACBE KO: 371790 unique reads)
TEAp bait
(WT: 1001934 unique reads,
EACBE KO: 978265 unique reads)
Trav17 bait
(WT: 874296 unique reads,
EACBE KO: 474563 unique reads)
INT2 bait
(WT: 1726677 unique reads,
EACBE KO: 967242 unique reads)
WT
KO
Frequency
3–way
4–way
5–way
6–way
7–way
8–way
b
Bait: Eα
Bait: TEAp
Bait: Trav17
Bait: INT2
Normalized interaction
8e+05
6e+05
4e+05
2e+05
0e+00
53.8
53.9
54.0
54.1
54.2
54.3 (Mb)
Trav17
Trav21
Trdv2-2
INTs
TEAp
Eα
c
Difference
0e+00
−1e+05
−2e+05
−3e+05
d
(Bait)
53.74Mb
54.24Mb
p < 0.001
p = 0.033

Figure S3 Related to Figure 3a and 3b

Bait-SOI plots illustrating the co-occurrence contacts of sequences in the locus with the combination of the E_{α} bait and the sliding windows (4kb bin and 2kb step) of SOIs from TEAp to *Traj18*. The green line represents the observed co-occurrence frequencies of sequences, while the gray line represents the expected co-occurrence frequencies (mean ± s.d.) across the locus. The z-scores indicating the significance of enrichment or lack of a given site are displayed in the bottom rectangles. Dark blue color indicates significant enrichment, while dark red color indicates a significant lack of a given site.

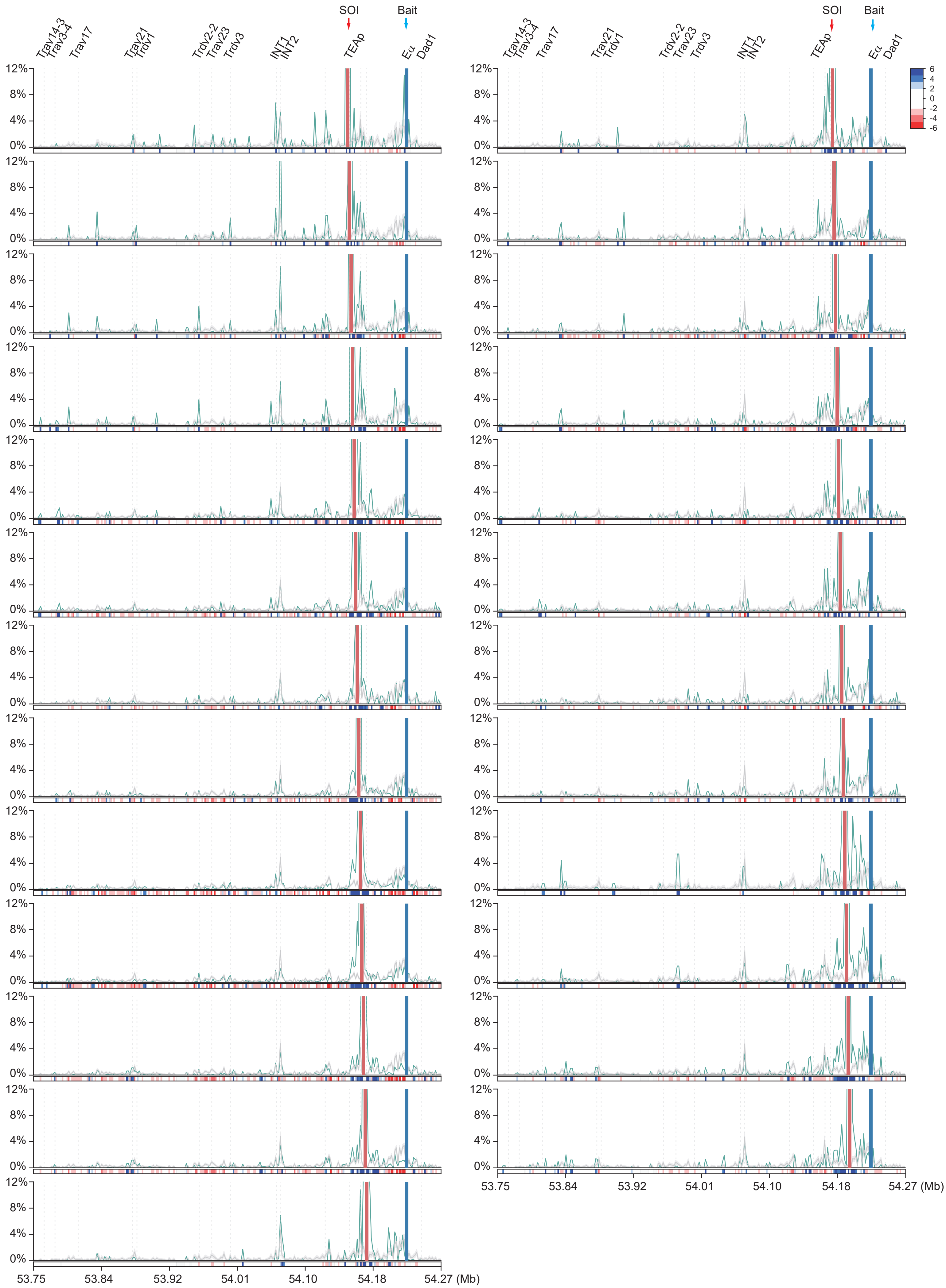

SOI
Bait
Trav14-3
Trav3-4
Trav17
Trav21
Trdv1
Trdv2-2
Trav23
Trdv3
INT1
INT2
TEAp
Eα
Dad1
12%
8%
4%
0%
6
4
2
0
-2
-4
-6
53.75
53.84
53.92
54.01
54.10
54.18
54.27 (Mb)

Figure S4 The E_α promotes the proximity of the proximal J_α genes to the V_α region in DP thymocytes.

a) 3C-HTGTS signal counts (teal) of the 3' portion of the *Tcra*-*Tcrd* locus, from the E_α -containing fragment (bait2). The normalized CTCF ChIP-seq profile (blue) in DP cells is shown below the 3C-HTGTS track.

b) Heatmap showing three-way contacts of the 3' portion of the *Tcra*-*Tcrd* locus. Resolution: 5kb; Coordinates (mm10): chr14:53740000-54300000

c) Bait-SOI plot displaying the co-occurrence contacts of sequences in the locus with the combination of the E_α bait and the SOI of the *Traj61*-to-*Traj56* region.

d) and **e**) Bar graphs displaying the co-occurrence unique read counts in the combination of the E_α bait2 and the SOI of (**d**) the sequence of the proximal J_α region or (**e**) the sequences containing *Trav3-4*, *Trav12-4*, *Trav17*, *Trav19* , or *Trdv2-2*, respectively. The third points represent the five V_α genes or the proximal J_α region. The green bars represent the enrichment of the given third site in bait-SOI co-occurrence, and the gray bars represent the enrichment of the given third site in bait contacts without SOI. The data represent mean ±s.d.

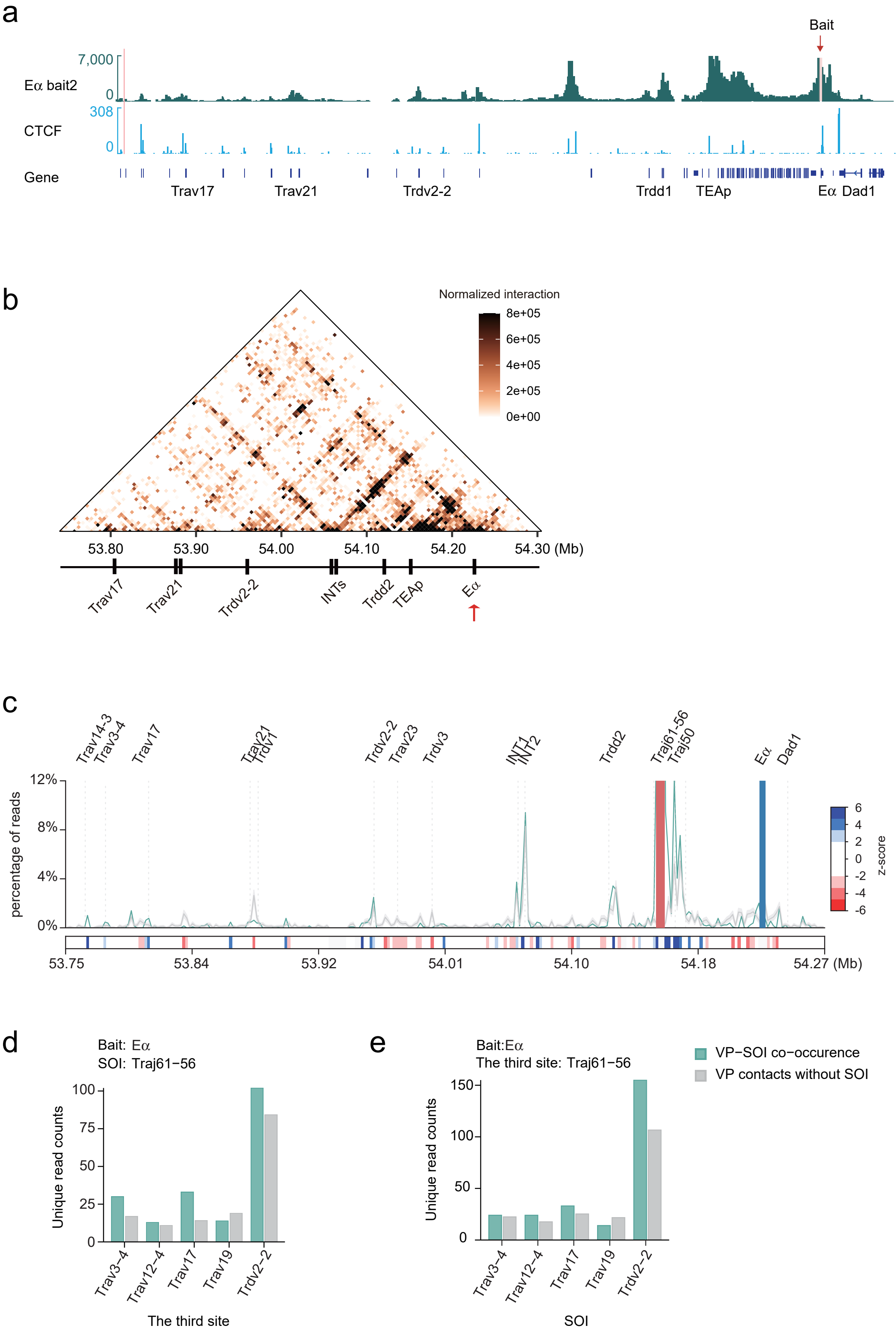
a
Bait
Eα bait2
7,000
0
CTCF
308
0
Gene
Trav17
Trav21
Trdv2-2
Trdd1
TEAp
Eα
Dad1
b
Normalized interaction
8e+05
6e+05
4e+05
2e+05
0e+00
53.80
53.90
54.00
54.10
54.20
54.30 (Mb)
Trav17
Trav21
Trdv2-2
INTs
Trdd2
TEAp
Eα
c
Trav14-3
Trav3-4
Trav17
Trav21
Trdv1
Trdv2-2
Trav23
Trdv3
INT1
INT2
Trdd2
Traj61-56
Traj50
Eα
Dad1
percentage of reads
12%
8%
4%
0%
53.75
53.84
53.92
54.01
54.10
54.18
54.27 (Mb)
z-score
6
4
2
0
-2
-4
-6
d
Bait: Eα
SOI: Traj61−56
Unique read counts
100
75
50
25
0
Trav3−4
Trav12−4
Trav17
Trav19
Trdv2−2
The third site
e
Bait:Eα
The third site: Traj61−56
Unique read counts
150
100
50
0
Trav3−4
Trav12−4
Trav17
Trav19
Trdv2−2
SOI
VP−SOI co−occurence
VP contacts without SOI

Figure S5 The proximal J_α region is disfavored in contacts with the combination of the E_α bait and SOIs of V genes.

a) Bait-SOI plots displaying the co-occurrence contacts of sequences in the locus with the combination of the E_α bait and the SOIs of V_α genes. The green line represents the observed co-occurrence frequency of sequences, and the gray line represents the expected (mean ± s.d.) co-occurrence frequency of sequences across the locus.

b) Heatmap showing the z-score landscape of sequences in the locus in co-occurrence contacts with the combination of the E_α bait and the SOIs of V genes. Rectangles in the heatmap represent the value of z-score.

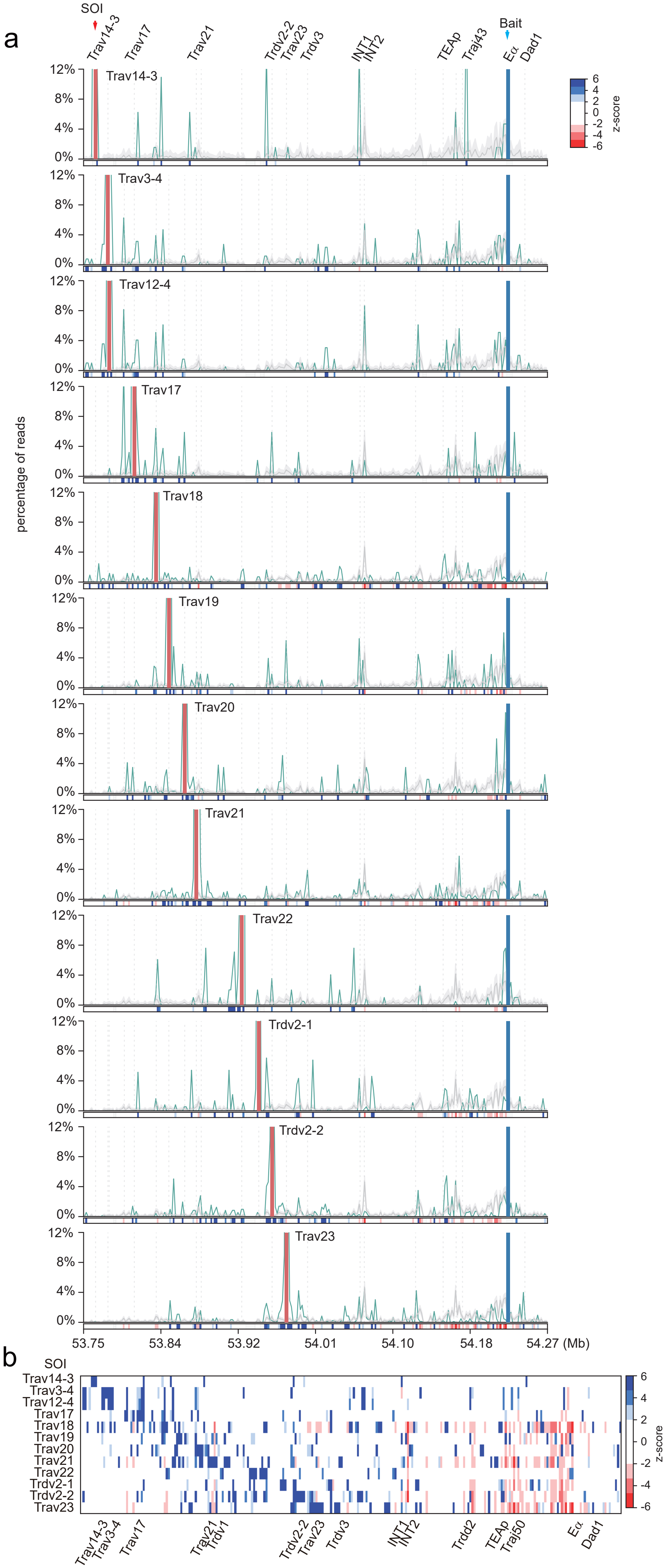
a
SOI
Bait
Trav14-3
Trav17
Trav21
Trdv2-2
Trav23
Trdv3
INT1
INT2
TEAp
Traj43
Eα
Dad1
percentage of reads
12%
8%
4%
0%
Trav14-3
Trav3-4
Trav12-4
Trav17
Trav18
Trav19
Trav20
Trav21
Trav22
Trdv2-1
Trdv2-2
Trav23
z-score
6
4
2
0
-2
-4
-6
53.75
53.84
53.92
54.01
54.10
54.18
54.27 (Mb)
b
SOI
Trav14-3
Trav3-4
Trav12-4
Trav17
Trav18
Trav19
Trav20
Trav21
Trav22
Trdv2-1
Trdv2-2
Trav23
Trav14-3
Trav3-4
Trav17
Trav21
Trdv1
Trdv2-2
Trav23
Trdv3
INT1
INT2
Trdd2
TEAp
Traj50
Eα
Dad1

Figure S6 Related to Figure 4b

Bait-SOI plots displaying co-occurrence contacts of sequences in the locus with the combination of the TEAp bait and the SOIs of the V genes. The green line represents the observed co-occurrence frequency of sequences, and the gray line the expected (mean ± s.d.) co-occurrence frequency of sequences. The z-scores are shown in the bottom rectangles, with dark blue indicating significant enrichment and dark red indicating significant lack of a given site.

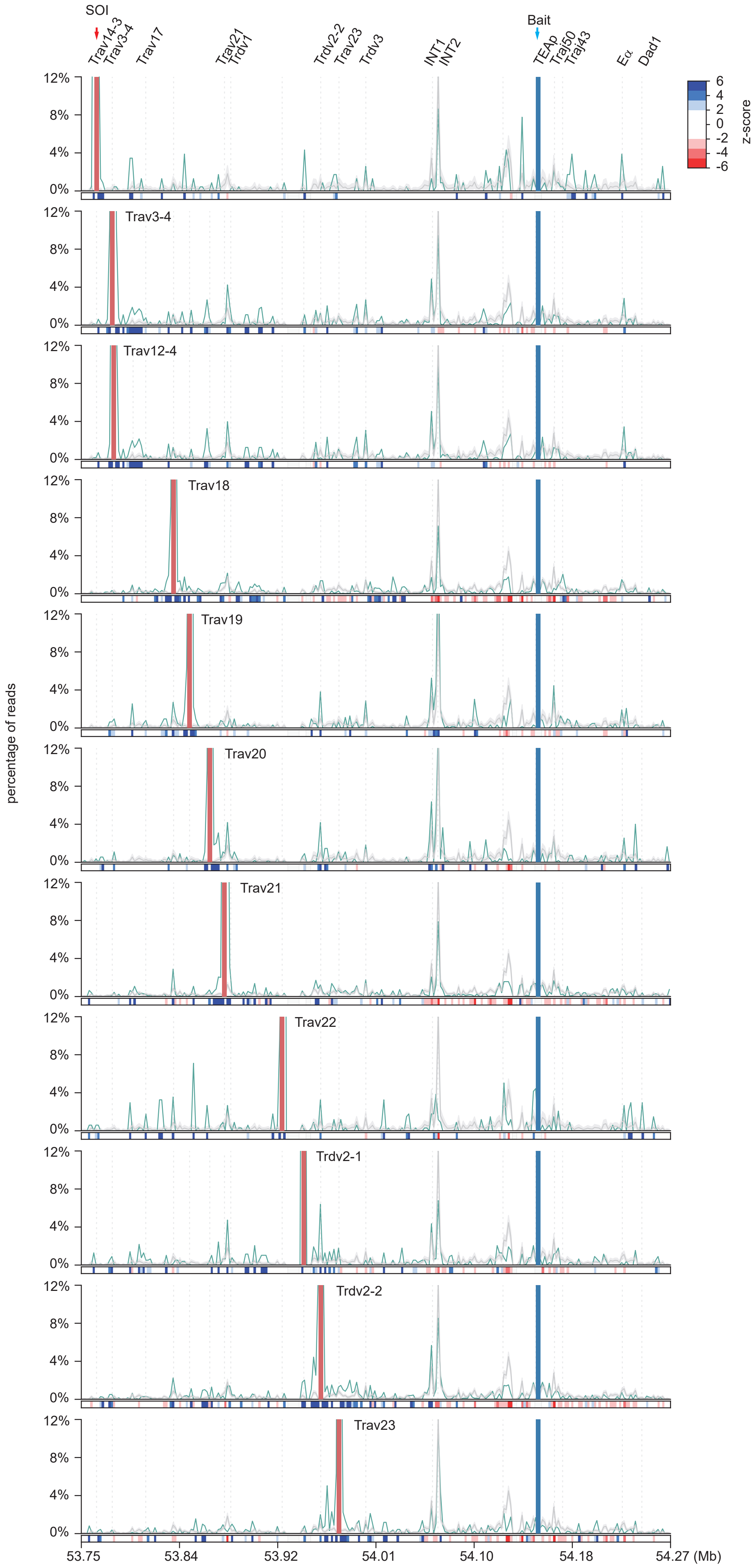

SOI
Bait
Trav14-3
Trav3-4
Trav17
Trav21
Trdv1
Trdv2-2
Trav23
Trdv3
INT1
INT2
TEAp
Traj50
Traj43
Eα
Dad1
z-score
6
4
2
0
-2
-4
-6
percentage of reads
12%
8%
4%
0%
Trav3-4
Trav12-4
Trav18
Trav19
Trav20
Trav21
Trav22
Trdv2-1
Trdv2-2
Trav23
53.75
53.84
53.92
54.01
54.10
54.18
54.27 (Mb)

Figure S7 Related to Figure 4f

Bait-SOI plot displaying co-occurrence contacts of sequences in the locus with the combination of the *Trav17* bait and the SOI sliding windows (4kb bin and 2kb step) from TEAp to Traj18. The green line represents the observed co-occurrence frequency of sequences, and the gray line represents the expected (mean ± s.d.) co-occurrence frequency of sequences.

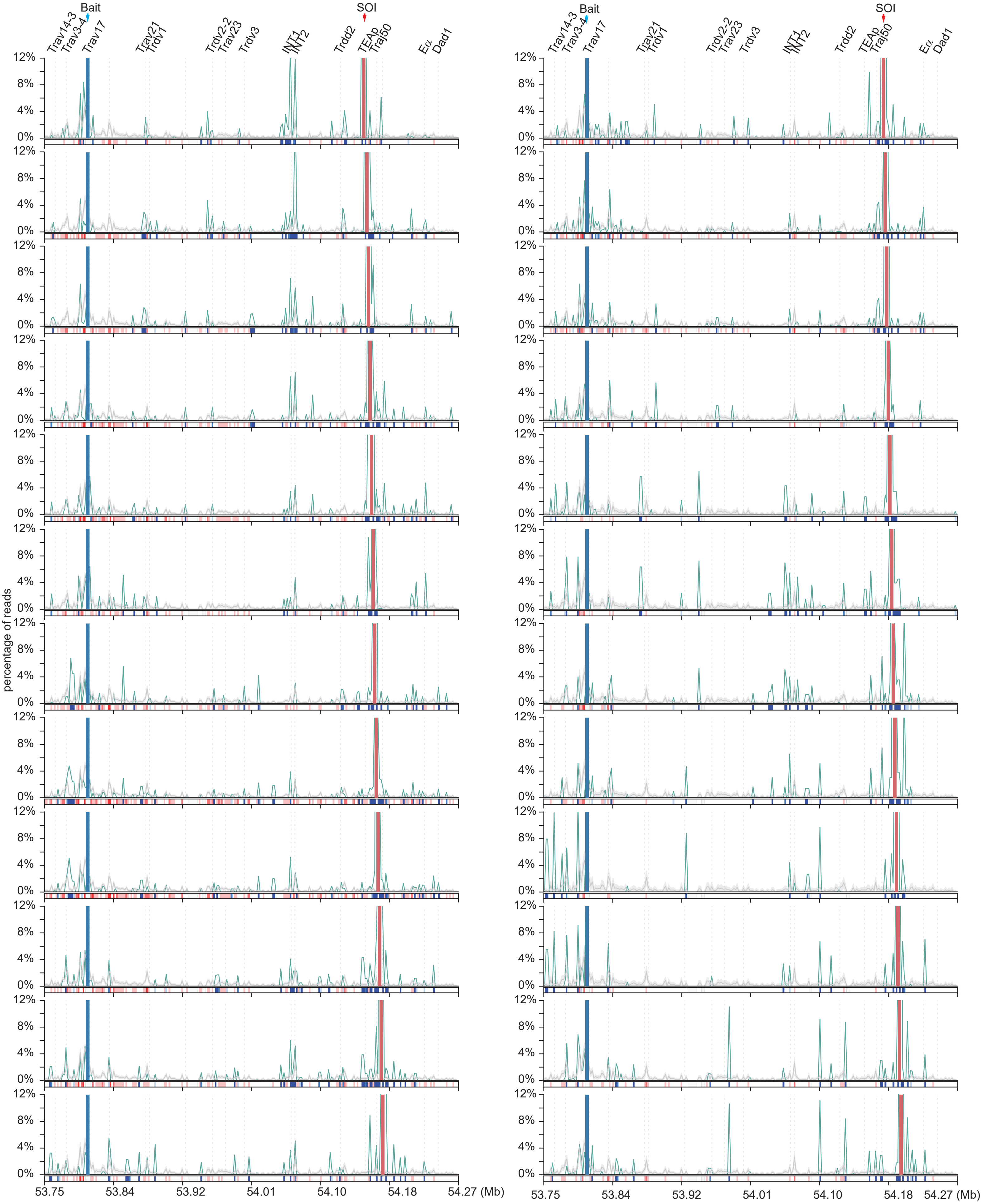

Bait
SOI
Trav14-3
Trav3-4
Trav17
Trav21
Trdv1
Trdv2-2
Trav23
Trdv3
INT1
INT2
Trdd2
TEAp
Traj50
Eα
Dad1
12%
8%
4%
0%
percentage of reads
53.75
53.84
53.92
54.01
54.10
54.18
54.27 (Mb)

Figure S8 Related to figure 5

a) 3C-HTGTS pair-wise chromatin interactions profiles of the 3' portion of the *Tcrα-Tcrd* locus in anti-CD3-induced DP thymocytes of *Rag1*$^{-/-}$ (WT) (teal) and EACBE$^{-/-}$ *Rag1*$^{-/-}$ (KO) (red) mice. Bait: *Trav17* or TEAp. Normalized CTCF, Rad21 and Nipbl ChIP-seq profiles in DP cells are displayed below.

b) Line plot displaying significant differences in pairwise chromatin interactions. The 4C-ker program was used for statistical analysis. Bait: *Trav17* (top) or TEAp (bottom). The analysis was performed with three independent experimental replicates. Filled circles in the line plot highlight interactions with statistically significant differences ($P < 0.05$).

c) Heatmap (Left) and subtraction heatmap (right) showing three-way contacts in the *Trav17* bait in anti-CD3-induced DP thymocytes of *Rag1*$^{-/-}$ (WT) and EACBE$^{-/-}$ *Rag1*$^{-/-}$ (KO) mice. Statistically significant different interactions are highlighted with black circles ($P < 0.001$). Gene annotation is shown below the heatmaps. Resolution: 5kb; Coordinates(mm10): chr14:53740000-54300000.

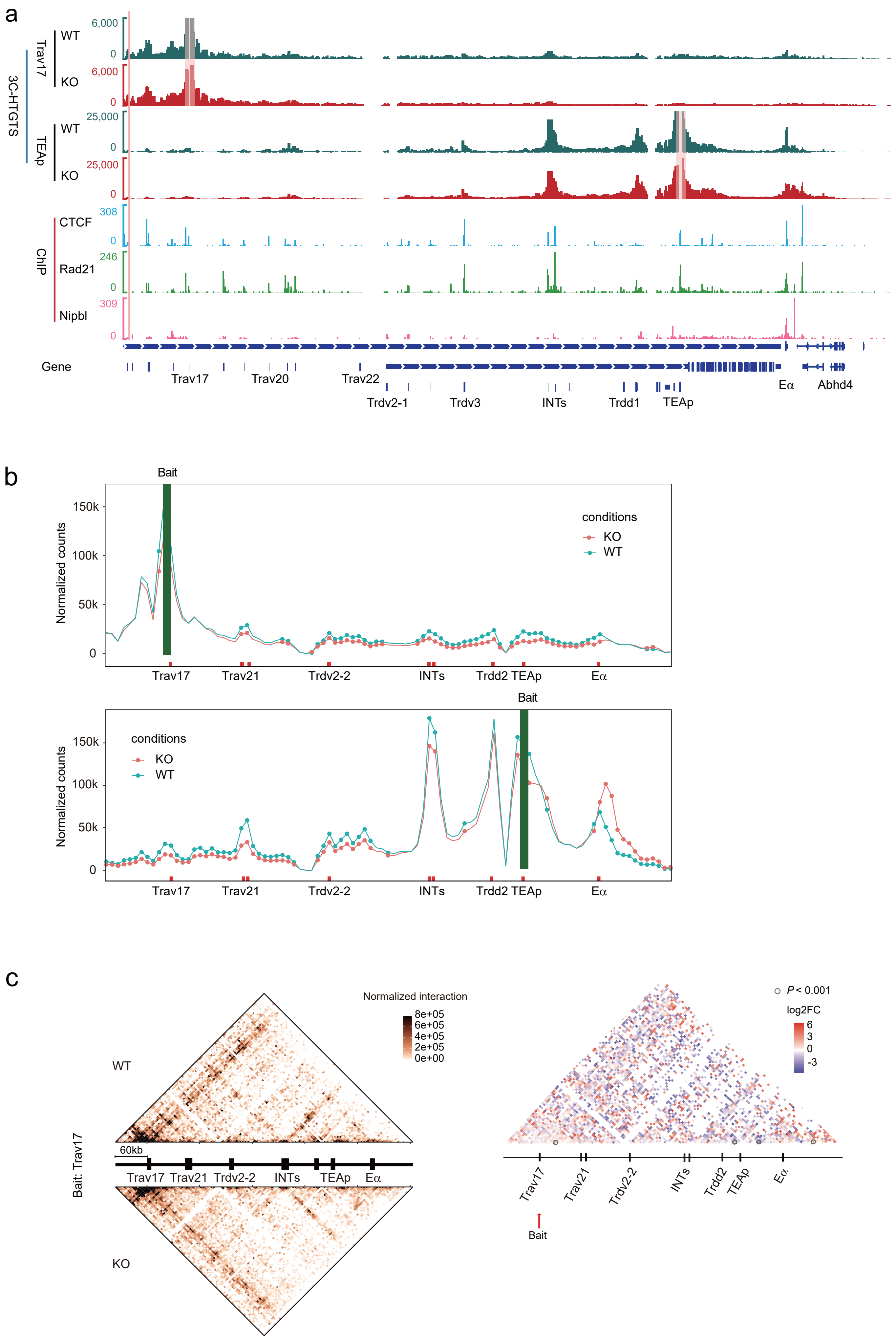

a
3C-HTGTS
Trav17
WT
KO
TEAp
WT
KO
6,000
0
6,000
0
25,000
0
25,000
0
ChIP
CTCF
Rad21
Nipbl
308
0
246
0
309
0
Gene
Trav17
Trav20
Trav22
Trdv2-1
Trdv3
INTs
Trdd1
TEAp
Eα
Abhd4
b
Bait
conditions
KO
WT
Normalized counts
150k
100k
50k
0
Trav17
Trav21
Trdv2-2
INTs
Trdd2 TEAp
Eα
c
Normalized interaction
8e+05
6e+05
4e+05
2e+05
0e+00
WT
KO
Bait: Trav17
60kb
Trav17 Trav21 Trdv2-2 INTs TEAp Eα
P < 0.001
log2FC
6
3
0
-3
Trav17
Trav21
Trdv2-2
INTs
Trdd2
TEAp
Eα
Bait
